# Unveiling the Fundamental Principles of Reconfigurable Resistance States in Silver/Poly(Ethylene Glycol) Nanofluids

**DOI:** 10.1002/advs.202505103

**Published:** 2025-06-26

**Authors:** Daniil Nikitin, Kateryna Biliak, Mariia Protsak, Blessing Adejube, Suren Ali‐Ogly, Kateřina Škorvanková, Veronika Červenková, Ronaldo Katuta, Marco Tosca, Jan Hanuš, Zulfiya Černochová, Peter Černoch, Petr Štěpánek, Oleksandr Boiko, Paulina Szymoniak, Andreas Schönhals, Franz Faupel, Hynek Biederman, Alexander Vahl, Andrei Choukourov

**Affiliations:** ^1^ Faculty of Mathematics and Physics Department of Macromolecular Physics Charles University V Holešovičkách 2 Prague 18000 Czech Republic; ^2^ Chair for Multicomponent Materials Department of Material Science Faculty of Engineering Kiel University Kaiserstraße 2 D‐24143 Kiel Germany; ^3^ ELI Beamlines Facility Extreme Light Infrastructure ERIC Za Radnicí 835 Dolni Brezany 25241 Czech Republic; ^4^ Institute of Macromolecular Chemistry CAS Heyrovského nám. 2 Prague 16206 Czech Republic; ^5^ Lublin University of Technology Department of Electrical Engineering and Superconductivity Technologies Nadbystrzycka St. 38A Lublin 20–618 Poland; ^6^ Bundesanstalt für Materialforschung und ‐prüfung (BAM) Unter den Eichen 87 12205 Berlin Germany; ^7^ Institut für Chemie Technische Universität Berlin Straße des 17. Juni 135 10623 Berlin Germany; ^8^ Kiel Nano, Surface, and Interface Science, KiNSIS Kiel University Christian‐Albrechts Platz 4 24118 Kiel Germany; ^9^ Leibniz Institute for Plasma Science and Technology Felix‐Hausdorff‐Str. 2 17489 Greifswald Germany

**Keywords:** bio‐inspired electronics, silver/poly(ethylene glycol) nanofluid, resistive switching, initialization, conductivity mechanism, molecular mobility of the polymer

## Abstract

Developing novel memristive systems aims to implement key principles of biological neuron assemblies – plasticity, adaptivity, and self‐organization – into artificial devices for parallel, energy‐efficient computing. Solid‐state memristive devices, such as crossbar arrays and percolated nanoparticle (NP) networks, already demonstrate these properties. However, closer similarity to neural networks is expected from liquid‐state systems, including polymer melts, which remain largely unexplored. Here, the resistive switching in silver/poly(ethylene glycol) (Ag/PEG) nanofluids, prepared by depositing gas‐aggregated Ag NPs into PEGs of varying molecular mass, is investigated. These systems form long‐range conductive NP bridges with reconfigurable resistance states in response to an electric field. The zeta‐potential of Ag NPs and molecular mobility of PEG determine the prevalence of low resistance (ohmic) state, high resistance states (poor conductance) or intermediate transition states governed by space‐charge‐limited conduction or electron tunneling. The occurrence of these states is given by the interparticle gaps, which are determined by the conformation of PEG molecules adsorbed on the NPs. It is presented, for the first time, an equivalent circuit model for the Ag/PEG system. These findings pave the way to adopt polymer melts as matrices for neuromorphic engineering and bio‐inspired electronics.

## Graphical abstract

The electrophoretic reorganization of Ag nanoparticles within a poly(ethylene glycol) leads to the formation of conductive bridges with reconfigurable resistance states. Diverse conduction characteristics of the bridge range between ohmic conduction, nonlinear tunneling, or space charge‐limited conduction, and high resistance states. The molecular mobility of bulk and adsorbed PEG depends on chain length and impacts the prevalence of resistance states.

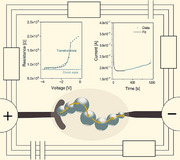

## 1 Introduction

The rising computational demands related to artificial intelligence via artificial neural networks and the associated surge in energy consumption result in significant challenges in the development of novel computational technologies. While conventional computing architectures excel at high‐precision and ultra‐fast sequential processing, the highly parallel nature of processing in common operations in artificial neural networks, such as inference, imposes growing demands on energy consumption.^[^ ^1^, ^2^ ^]^ In particular, the current computing paradigm uses von Neumann architecture and relies on continuous data transfer between a physically separated memory and processing units, resulting in a bottleneck.^[^ ^3^, ^4^ ^]^ In contrast, biological neural networks are self‐organized, highly interconnected, and remarkably adaptive, capable of performing complex tasks, such as image or speech recognition, with minimal power usage, often consuming only a few watts.^[^ ^5^ ^]^ To complement conventional computing and to meet the demands of efficient, highly parallel computing, neuromorphic engineering takes inspiration from biological neural networks and the electrical properties of their building units (neurons and synapses), implementing brain‐inspired information pathways in artificial electronic devices.^[^ ^6^ ^]^

A common approach to mimic synaptic properties can be found in reconfigurable resistance states of memristive devices. These are commonly two‐terminal devices that can switch their resistance between two or more resistance states under the influence of an external electric field, a phenomenon known as resistive switching.^[^ ^7^ ^]^ This switching behavior allows memristive devices, as electronic synapses, to be functionally similar to biological synapses, which transmit information through changes in ionic conductivity. Various synapse‐like properties, such as spike timing‐dependent plasticity, long‐term potentiation, and depression, have been demonstrated in a wide range of memristive devices. While resistive switching has been extensively studied in solid‐state devices, in the last decade, in the search for pathways toward brain‐inspired hardware for information processing, a series of complementary approaches arose, in particular relying on an expansion of the materials toolbox beyond conventional solid‐state devices toward liquid media,^[^ ^8^ ^]^ organic materials^[^ ^9^ ^]^ and nanogranular matter.^[^ ^10^ ^]^

In solid‐state systems, resistive switching typically traces back to phenomena such as amorphous/crystalline transitions in phase‐change memories or conductive bridge formation through the reversible metallic filament growth/dissolution in electrochemical metallization cells, or oxygen vacancy migration in valence‐change memories. Most solid‐state memristive devices currently use thin film stacks in metal‐insulator‐metal (MIM) configuration, particularly promising in crossbar arrays for efficient operation in highly parallel tasks such as vector‐matrix multiplication.^[^ ^11^, ^12^ ^]^ Besides MIM memristive devices as electronic synapses, there is an increasing interest in mimicking further features of neural networks, such as self‐organized network growth, distributed plasticity, criticality, hierarchy, and modularity. In solid‐state systems, these aspects have been addressed by bottom‐up approaches and self‐organization in percolated networks of metal nanowires and nanoparticles (NPs), which are capable of exhibiting collective resistive switching responses and avalanche criticality.^[^ ^10^, ^13^, ^14^, ^15^, ^16^, ^17^ ^]^ However, these nanogranular networks are to date confined to planar, quasi‐2D connection schemes.

In liquid state systems, mimicking of synaptic processes is approached by the transfer of signals via ionic processes, introducing further brain‐inspired features such as homeostasis and global connectivity through local gating^[^ ^18^ ^]^ or employing arrays of oscillatory reactions for probabilistic computing.^[^ ^19^ ^]^ Resistive switching in liquid media, such as through electric field‐guided redox wiring,^[^ ^8^ ^]^ shares similarities with conventional solid‐state MIM devices, but occurs on a larger spatiotemporal scale. This opens the possibility of introducing 3D conduction, robustness, modularity, hierarchical organization, and self‐organized ordering.^[^ ^20^ ^]^ In these devices, changes in conductivity are determined by ionic transport between two reservoirs filled with electrolyte or ionic liquid through micro‐ to nanoscale channels.^[^ ^21^, ^22^, ^23^ ^]^ Recently, angstrom‐sized channels (comparable to atomic dimensions) have been utilized to accelerate switching dynamics and achieve exceptionally low power consumption, as low as 2–23 fJ/spike for a single channel.^[^ ^24^ ^]^

Recently, resistive switching has also been reported in Ag NPs/poly(ethylene glycol) (PEG) nanofluids as solid‐liquid composite systems, with metal NPs being embedded into dielectric polymer melts.^[^ ^25^ ^]^ Here, Ag NPs act as conductive building blocks that move within a dielectric polymer melt under the influence of an electrical field, eventually forming a long‐range percolated NP bridge with a characteristic hysteresis in the current‐voltage (*I*–*V*), indicating resistive switching with reconfigurable resistance states. Polymer melts as host liquids provide exciting complexity in their molecular mobility. Multi‐length scale molecular motions occur inside a polymer, spanning from the microscopic level of bond vibrations (< 1 nm) to molecular motions of local segments and cooperative rearranging regions (1–10 nm) and further to the motion of the entire chain (1–10 nm). These motions cover broad time scales corresponding to 10^13^–10^−5^ Hz. If used as embedding matrices for metal NPs, polymer melts mediate the motion of NPs and influence resistive switching phenomena in percolated NP networks, as was shown for Ag NPs in PEG^[^ ^25^ ^]^ and other polymers.^[^ ^26^ ^]^

However, to date, it remains unexplored how the complex molecular mobility of polymer influences the dynamics of the initialization of long‐range conductive NP bridges and resistive switching, and whether the advantage may be taken to diversify the synaptic functionality of metal nanofluids. The current study aims to answer these questions by investigating Ag/PEG nanofluids prepared with PEGs of different chain lengths, with *operando* studies of initialization and resistive switching (*I–t* and *I*–*V*) correlated to research on the molecular dynamics of PEGs.

## 2 Results and Discussion

### 2.1 Forming of the Conductive Bridge in Different PEGs

**Figure 1:**
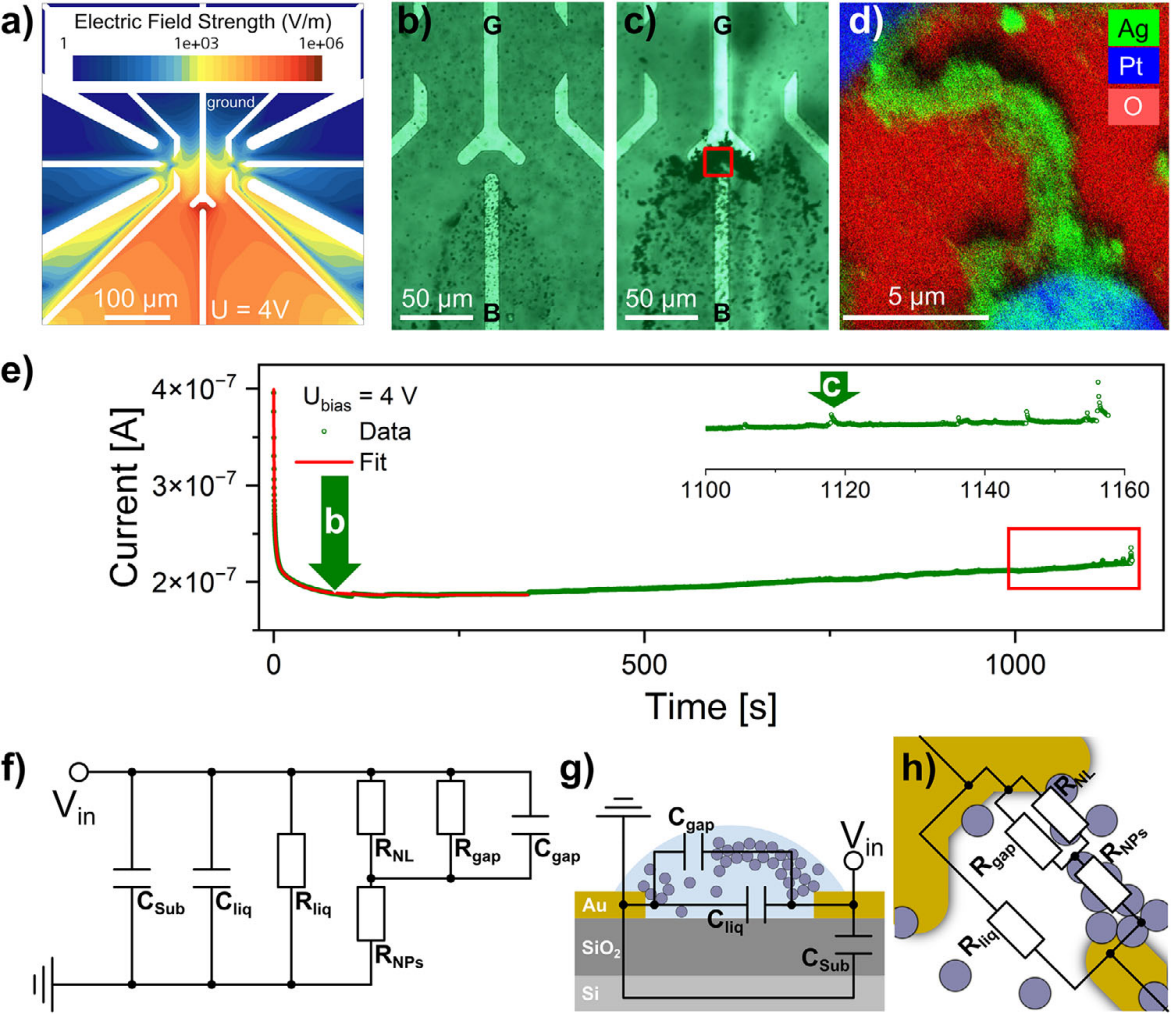
Electric field‐driven evolution of the agglomeration zone: a) 2D model of the electric field strength; b), c) optical micrographs of the Ag/PEG 400 nanofluid at the beginning of the conductive bridge growth and when the first spikes appeared; a constant voltage of +4 V was applied between the electrode B (biased) and G (grounded). The bridge of NPs connecting the Pt electrodes: d) EDX map, acquired in the zone represented by the red frame in Figure 1c. The corresponding SEM images are presented in the Supporting Information. Example of *I‐t* characteristic: e) data (green) with the fit of the initial part (red) representing the charging of capacitances contained in the system. Green arrows represent the time of the snapshots b) and c). f) Schematic equivalent circuit corresponding to the Ag/PEG nanofluidic system. g) The detailed representation of the droplet cross‐section with capacitive components and h) the top view with resistive components.

In previous work, the resistive switching phenomena in Ag/PEG nanofluids were adversely affected by the asymmetric field distribution, which increased the anisotropy in the system.^[^ ^25^ ^]^ To address this issue, we optimized the electrode configuration in this study by aligning two coaxial electrodes at the center of the substrate. This was done to achieve a symmetric electric field, as shown schematically in **Figures** **1**a and S1 (Supporting Information). A detailed characterization of the experimental cell is given in the Supporting Information. The simulated 2D map of the electric field distribution in Figure 1a shows a highly symmetrical field with a field strength of 10^6^ V m^−1^ in the interelectrode gap. This field strength agrees with the values reported earlier in ref. [25] The field strength is highest near the biased electrode and decreases with distance. This electrical field was achieved with a lower applied voltage of *V_in_* = +4 V by reducing the interelectrode gap from 33 µm used in the previous setup to 12 µm employed in the experiments reported here. Furthermore, the reduction in interelectrode distance significantly lowers the risk of electrochemical decomposition of adsorbed water, which can lead to bubble formation. As reported in ref. [25], such decomposition effects were observed only at voltages exceeding ±6 V. In the present study, the formation of conductive pathways and the onset of stable resistive switching occur at lower bias voltages of ±4 V, which is below the threshold for water electrolysis. Therefore, no signs of electrochemical decomposition were observed under our operating conditions.

Upon application of a constant potential of *V_in_* = +4 V, Ag NPs and their agglomerates begin to migrate toward the biased electrode. Video S1 shows the movement in the Ag/PEG400 nanofluid. It can be concluded that Ag NPs in the nanofluid are negatively charged and move through electrophoresis. The negative charge can be attributed to different mechanisms. Previous DFT calculations modeled the interaction of a diethylene glycol (DEG) molecule (the shortest homologue of PEG) with the Ag surface at monodentate coordination (one oxygen of the hydroxyl group of DEG binds to Ag) and bidentate coordination (two oxygen of the hydroxyl groups of DEG bind to Ag), revealing that a negative charge is formed on the adsorbed DEG molecule due to the redistribution of the electronic density from Ag to O atoms.^[^ ^27^ ^]^ Furthermore, the adsorption of anionic species, primarily hydroxide ions (OH⁻), on the NP surface may also take place. These ions may originate from trace amounts of water absorbed from ambient air, the effect which is well studied for metal NPs dispersed in polar hygroscopic media^[^ ^28^, ^29^, ^30^ ^]^ or from the dissociation of the hydroxyl groups of PEG.

The Ag NPs accumulate around the biased electrode and in the interelectrode region, forming an agglomeration zone, as shown in Figure 1b. Similar behavior was observed for the Ag/PEG200 and Ag/PEG600 nanofluids. The negative charge of NPs moving toward the positive electrode is confirmed by the negative *ξ*‐potential values measured by electrophoretic light scattering (**Table** **1**). The agglomeration zone expands over time, eventually covering the entire inter‐electrode gap and creating a bridge. This process is similar to the forming step in classic memristive devices. An optical microscopy image of the bridge is provided in Figure 1c, and Figure 1d depicts the energy‐dispersive X‐ray analysis (EDX) map of the bridge area. This map highlights the distribution of the elements of interest: silver, platinum, and oxygen. The distributions of silicon and carbon, as well as the corresponding scanning electron microscopy (SEM) images, can be found in Figure S2 (Supporting Information). The Ag‐based filament connecting the Pt electrodes comprises Ag NPs. The EDX map reveals that oxygen surrounds the conductive bridge, which is due to SiO_2_ on the surface of the substrate. It also indicates the absence of silver oxide in the Ag NPs, which would decrease their electrical conductivity.

**Table 1:** The results on the ξ‐potential and electrophoretic mobility of Ag NPs in different PEGs. The concentration of nanofluids was 50 µg mL^−1^.

M_n_ of PEG [g mol^−1^]	Temperature [⁰C]	*ξ*‐potential [mV]	Electrophoretic mobility [$\frac{\mu m\times cm}{V\times s}$]
200		− 30	− 6 × 10^−3^
400	25	− 27	− 2 × 10^−3^
600		− 19	− 1 × 10^−3^

The initialization and formation of the long‐range bridge were monitored through the *I*‐*t* measurements as well. The role of the initialization in resistive switching and its similarities and differences to electroforming in solid‐state memristive devices are discussed in Note S6 (Supporting Information). The *I‐t* curve corresponding to Video S1 (Supporting Information) is shown in Figure 1e, where green arrows indicate the times when the snapshots in Figure 1b,c were taken. When the voltage of a *V_in_* = +4 V was applied to the biased electrode, the current through the nanofluid decreased exponentially, stabilizing after ≈150 s. This decrease can be attributed to the internal capacitance related to the permittivity of PEG, which causes electric charges to accumulate at the electrode surfaces (electrode polarization).^[^ ^31^ ^]^ This is supported by the exponential decay of the current, which stabilizes when internal capacitive components are fully charged. After ≈200 s, NPs and NP agglomerates are attracted to the positively biased electrode, and an agglomeration zone is formed, reducing the effective interelectrode distance. This leads to a progressive increase in local electric field strength, enhancing ionic conduction through PEG and causing a gradual rise in current. The increase in current reflects a gradual deviation from the base resistance of the nanofluid (*R_liq_*) in its pristine state. Finally, the characteristic spikes appeared at ≈1100 s in the *I‐t* characteristic (Figure 1e). In solid‐state percolated NP networks, spikes in *I‐t* characteristics are typically attributed to the stochastic formation and disruption of atomic‐scale metal filaments within the interparticle gaps.^[^ ^17^, ^32^, ^33^ ^]^ However, in our study, the current spikes did not reach values much higher than the background current level of the host PEG. Therefore, we hypothesize that the observed spiking behavior corresponds to an under‐percolated long‐range NP bridge. In this state, there are sufficient NPs and NP agglomerates within the interelectrode gap to create a long‐range NP bridge, which results in a deviation from *R_liq_*. However, the interparticle distances still keep the nanofluid in its high resistance state (HRS). Furthermore, it must be pointed out that the temporal resolution of the measurement setup limited the observability of the formation and dissolution of individual atomic‐scale filaments, which would be expected in solid‐state NP networks at the percolation threshold. With its clear deviation from *R_liq_*, the occurrence of spiking events can be treated as a termination criterion for the initialization process (a more detailed discussion can be found in the Note S1, Supporting Information). In order to quantify the initialization of the long‐range NP bridge, the time integral of the current response under DC bias until the occurrence of the first characteristic spikes can be considered to calculate the energy consumed during this step. In case of the *I‐t* curve shown in Figure 1e, an initialization energy of 871 µJ is obtained for a long‐range NP bridge across an electrode spacing of 12 µm.

Fitting the initial region of the *I‐t* characteristic with an exponential function provided insight into the electrophysical characteristics of the nanofluidic system. For Ag/PEG nanofluids, the best fit was achieved using a three‐parameter exponential function, as presented in Equation 1 (R‐squared > 0.99), while only two components were sufficient to fit the *I‐t* data of unfilled PEGs with the same accuracy (see Figures S3 and S4, Supporting Information).

(1) $$\begin{array}{c} i=i_{0}+C_{1}\times \exp\left(-\frac{t}{\tau_{1}}\right)+C_{2}\times \exp\left(-\frac{t}{\tau_{2}}\right)+C_{3}\times \exp\left(-\frac{t}{\tau_{3}}\right) \end{array}$$

In this equation, *i_0_* represents the base current, which can be converted to *R_liq_* using Ohm's law $R_{liq}=\frac{V_{in}}{i_{0}}\;$. *C_1_, C_2_* and *C_3_* correspond to different intrinsic capacitive components, which are charged with the time constants *τ_1_, τ_2_* and *τ_3_*, respectively.

The estimated fitting parameters are presented in **Table** **2**. The *R_liq_* increases from PEG200 to PEG600. Here, *R_liq_* directly represents the resistance of the host liquid, and it is related to the drift motion of charge carriers (ions) via a slave mechanism triggered by segmental fluctuations. The relaxation time for segmental motions increases from PEG200 to PEG600; therefore, the conductivity decreases from PEG200 to PEG600. More details about the molecular mobility of PEGs will be given later; here, it should be noted that, in the intrinsic state of the Ag/PEG, the separation of the NPs and agglomerates is too large to have a significant impact on *R_liq_*. The values of *C_1_* and *C_2_* are approximately of the same order of magnitude. However, the time constants have substantially different values. *C_3_* has the lowest value but the longest time constant.

**Table 2:** The physicochemical and electrophysical characteristics of Ag/PEG nanofluidic systems: tabulated and determined via the fitting of I‐t characteristics.

M_n_ of PEG [g mol^−1^]	Dynamic viscosity at 20⁰C (Pa·s) ^[^ ^34^ ^]^	Static permittivity at 25⁰C^[^ ^35^ ^]^	R_liq_ [MΩ]	C_1_ [nF]	τ_1_ [s]	C_2_ [nF]	τ_2_ [s]	C_3_ [nF]	τ_3_ [s]
200	0.063	22.1	20.8 ± 11.4	260 ± 150	0.48 ± 0.16	110 ± 60	8.0 ± 3.9	81 ± 40	70.0 ± 51.1
400	0.129	17.3	31.4 ± 9.9	170 ± 60	0.18 ± 0.02	120 ± 20	2.6 ± 0.8	60 ± 15	23.7 ± 6.0
600	0.165	14.3	40.9 ± 5.6	82 ± 21	0.23 ± 0.03	49 ± 15	2.8 ± 0.8	41 ± 18	19.8 ± 5.2

We developed an equivalent circuit model for the nanofluidic system, as shown in Figure 1f–h. By analyzing the capacitances and time constants obtained from the *I‐t* characteristics of PEGs without NPs (see Table S1, Supporting Information), we were able to assign physical meanings to different capacitive components. The capacitance *C_1_* corresponds to the gaps between Ag NPs that are distributed within the nanofluid in the interelectrode gap. This capacitance has the shortest time constant *τ_1_* ∼ 1 s due to the large difference in size between the NPs and the bulk electrodes. Component *C_2_* is assigned to the contact between the electrodes through the PEG. It exhibits a value similar to *C_1_* and is assumed to be proportional to the dielectric permittivity of the base liquid; therefore, it is decreasing from PEG200 to PEG600 (see Table 2). To support the relationship between *C_2_* and the capacitance of the host liquid, impedance spectroscopy measurements of PEG400 and Ag/PEG400 nanofluids were performed between 4 Hz and 10 MHz (see Figure S5, Supporting Information). The measured capacitance at 4 Hz reaches ≈100 nF, closely matching the *C_2_* values derived from the fitting of the *I‐t* data. This hypothesis is further supported by the values of the time constants *τ_2_* for the samples with and without NPs. Both are in the range of seconds, with slightly higher values for bare PEGs, which can be related to the lower overall conductivity of the liquid.

The parameter *C_3_* is likely related to capacitance due to the substrate, which is a silicon wafer with a thermal SiO_2_ layer. This is because its low value is consistent with the low dielectric permittivity of SiO_2_ (ε_SiO2_ = 3.9). *C_3_* has a long time constant, around tens of seconds, which is also confirmed by *C_3_* and τ_3_ estimated for the PEGs without NPs. As illustrated in Figure 1h, there are three resistance components: the base liquid resistance (*R_liq_*), the intrinsic resistance of Ag NPs (*R_NPs_*), and linear and non‐linear resistances within the gap between the growing bridge and grounded electrode (*R_gap_* and *R_NL_*). Overall, the electrophysical properties of the nanofluidic systems, specifically *R_liq_*, *C_1_* and *C_2_*, as well as *τ_1_* and *τ_2_* are strongly dependent on the type of base fluid. This suggests that the choice of PEG and its conductivity can be used to fine‐tune critical processes in resistive switching, such as the formation of a bridge between electrodes.

Viscosity, as well as the molecular mobility of the base fluid, significantly influences the diffusion of the NP. This conclusion can be deduced from the results of the measurements of the electrophoretic mobility of NPs, which decreases as the viscosity increases from PEG200 to PEG600 (see Table 1). The CFD simulations (Video S2, Supporting Information) confirmed that the diffusion of NPs is the fastest in PEG200 and the slowest in PEG600. The mobility of NPs affects the growth rate of the bridge, as was confirmed by analysis of the expansion rate of the aggregation zone (for details, see Figures S6 and S7. Supporting Information and the relevant text). A further increase in the molecular weight of the PEG matrix to 1000 g/mol could slow down the mobility of the NPs even more and create a quasi‐stable composite material due to a higher viscosity. Unfortunately, the viscosity of PEG1000 at room temperature is too high to allow for the direct loading of gas‐aggregated NPs into liquid, which is used for nanofluid synthesis in the current work. These findings demonstrate that the viscosity and molecular mobility of PEG directly influence the kinetics of the bridge formation, providing a tuning parameter for optimizing resistive switching applications. Markedly, once the bridge is formed, it demonstrates peculiar resistive switching phenomena as discussed in the following sections.

### 2.2 Analysis of Switching and the Mechanisms of Conductivity in Ag/PEG Nanofluids

**Figure 2:**
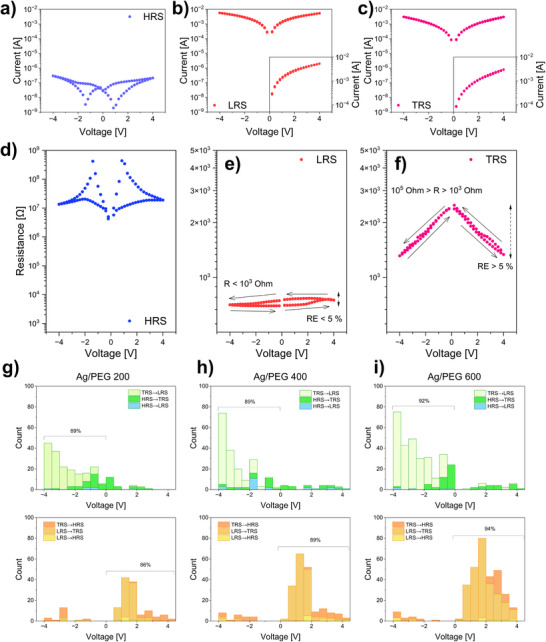
The selected *I*–*V* characteristics represent a) HRS, b) LRS, and c) TRS in the Ag/PEG400 nanofluids; d), e), and f) corresponding *R–V* characteristics. The histograms of SET voltage (green palette) and RESET voltage (orange palette) for g) Ag/PEG200, h) Ag/PEG400, and i) Ag/PEG600 were determined by the processing of *I*–*V* characteristics. Histograms are plotted in a cumulative manner.

After the onset of spiking instabilities in the *I–t* curves, the resistive switching in the nanofluidic system was measured by recording the cyclic *I*–*V* characteristics according to the protocol: 0 V→+4 V→−4 V→0 V. Examples of the *I*–*V* characteristics for Ag/PEG400 nanofluids are shown in **Figure** **2**a–c. In agreement with an earlier study,^[^ ^25^ ^]^ the Ag/PEG nanofluidic system undergoes multiple switching events between HRS, with a current below 10^−6^ A (Figure 2a), and low resistance state (LRS), with a current several orders of magnitude higher (Figure 2b). The comparison of the *I*–*V* characteristics reveals an ON/OFF ratio (switching ratio between the resistance state in HRS and LRS) of ≥10^3^. Additionally, it was observed that intermediate resistance states exist between the HRS and LRS, closer to the latter. From here on, the term transition resistance state (TRS) will be used to highlight its intermediate nature. Because of a subtle difference in the current between the LRS and TRS, it is not possible to distinguish unambiguously between them based on the *I–V* curves. However, for the sake of consistency, we have included them in Figure 2b,c (additional *I*–*V* curves are shown in Figures S8 and S9, Supporting Information). Reliable discrimination between different resistance states can be achieved by analyzing the average resistance $\bar{R}$ and its relative error *RE*, which is calculated from the *R‐V* characteristics obtained from the *I–V* curves using Ohm's law. Supporting Information provides details of these calculations. **Table** **3** shows the boundary conditions used to determine different resistance states.

**Table 3:** The conditions used for the determination of resistance states.

		$\bar{R}$		
		< 10^3^ Ω	10^3^ Ω ≤ $\bar{R}$ ≤ 10^5^ Ω	> 10^5^ Ω
*RE*	≥ 5%	TRS	TRS	HRS
	< 5%	LRS	TRS	HRS

The HRS is determined by the resistance in the MΩ range, which is characterized by the resistance of the host PEG (Figure 2d). As there is no ambiguity in determining the HRS from $\bar{R}$, the values of RE are not significant in this case. The LRS is characterized by a resistance in the sub‐kΩ range, with an invariance (RE < 5%) that indicates the Ohmic conductivity of the bridge in the percolated state (Figure 2e). In contrast, the TRS exhibits a resistance depending on the voltage that is higher than that of LRS but may approach the LRS values for higher voltages (Figure 2f). To differentiate between the LRS and TRS in this context, we utilize the increased volatility of the resistance in the TRS, resulting in RE > 5%. This suggests an un‐percolated network state for the TRS, where conducting aggregates are still disconnected, but the gap size is influenced by the mobility of the in‐gap PEG molecules. The additional R‐V characteristics demonstrating different resistance states and switching events between them are shown in Figure S10 (Supporting Information).

We analyzed the full set of the *I–V* characteristics to construct voltage distributions for SET and RESET events, considering all resistive switching. For SET switching, we defined three types of transitions: HRS→TRS, TRS→LRS, and direct HRS→LRS. For RESET switching, we included three other types: LRS→TRS, TRS→HRS, and direct LRS→HRS events. The resulting histograms are shown in Figure 2g–i. For all nanofluids, ≈90% of SET events occurred at the negative polarity of the biased electrode. The same percentage of RESET events occurred at the positive polarity. This polarity‐dependent prevalence of SET or RESET events was consistent across all switching types, as indicated by different colors in the histograms. Overall, for all nanofluids, TRS→LRS was the most common SET event, and LRS→TRS was the most frequent RESET event. Direct SET (HRS→LRS) and RESET (LRS→HRS), in contrast, were observed with the lowest probability in each nanofluid. The detailed analysis of the individual *R‐V* characteristics has allowed us to determine the values of the occurrence for each switching event, as well as the 10th, 50th, and 90th percentiles of the switching voltage. These values are summarized in Table S2 (Supporting Information).

**Figure 3:**
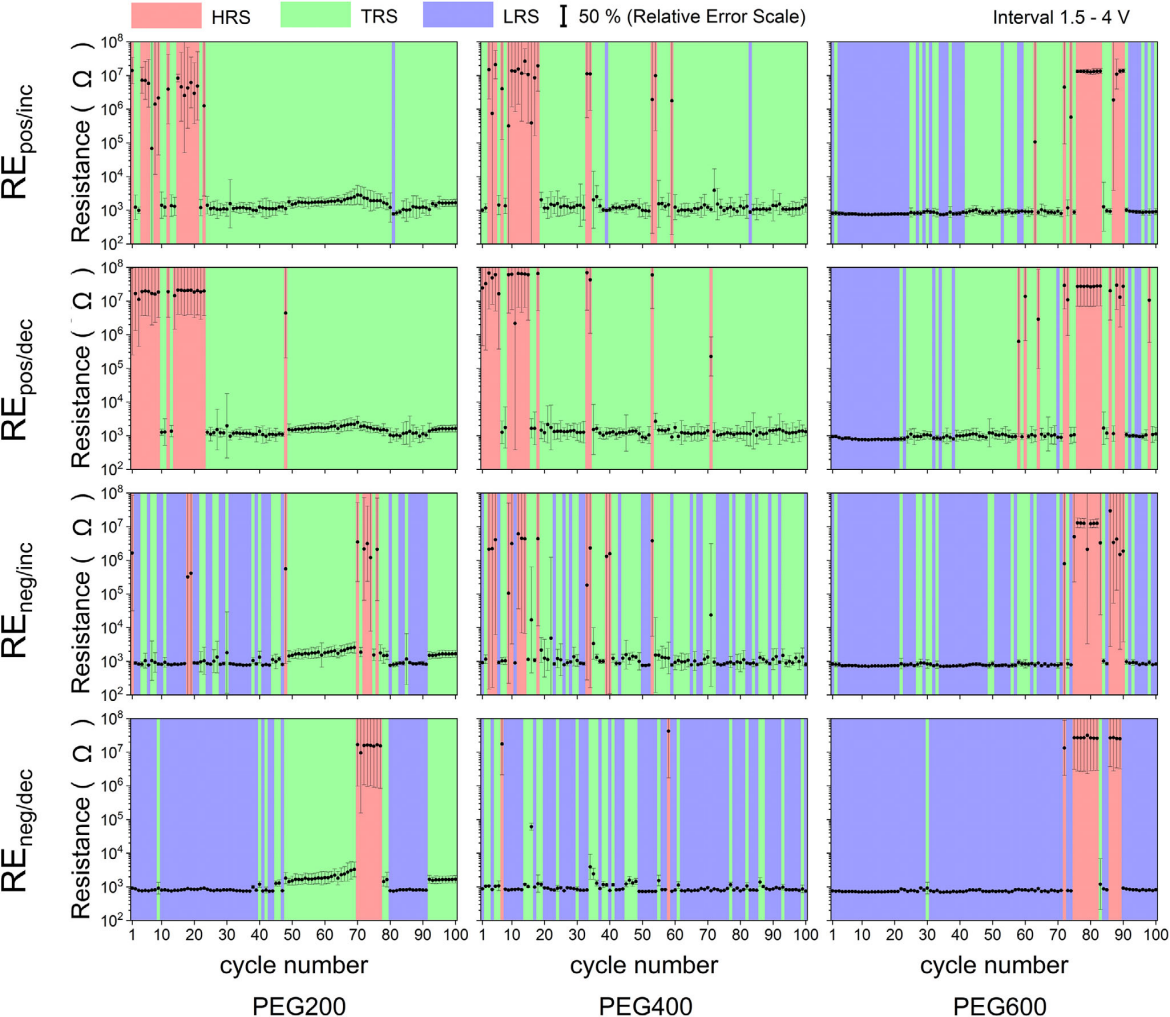
The distribution of individual resistance states on different branches of *I–V* characteristics during 100 cycles for each sample. The red color corresponds to HRS, green to TRS, and blue to LRS.

To further explore the above discussion, we plotted the distribution of individual resistance states on different branches of the *I–V* characteristics for 100 cycles measured for each nanofluid (**Figure** **3**). Although no distinct trend can be observed in the occurrence of the HRS, the prevalence of the LRS or TRS seems to depend on the polarity of the biased electrode and the molar mass of PEG. When the electrode has a positive polarity, NPs are attracted to it, depleting the inter‐electrode gap and contributing to the dissolution of a conductive bridge (RESET processes). The bridge typically remains in the HRS or TRS after this process. On the other hand, when the electrode has a negative polarity, the NPs are repelled, enriching the inter‐electrode gap and increasing the probability of conductive bridge formation. This is known as the SET process. A similar trend has been observed for different sets of 100 *I–V* characteristics, as shown in Figure S12 (Supporting Information).

To provide a physical interpretation of different resistance states, we consider a large reservoir of Ag NPs and aggregates (the bridge) created in the host matrix, which is mobile. Unlike solid‐state resistive switching, the mobile polymer matrix makes the bridge highly unstable, such that its resistance state is determined by the length scale of the inter‐agglomerate gaps filled with PEG as follows.

*HRS*

**Figure 4:**
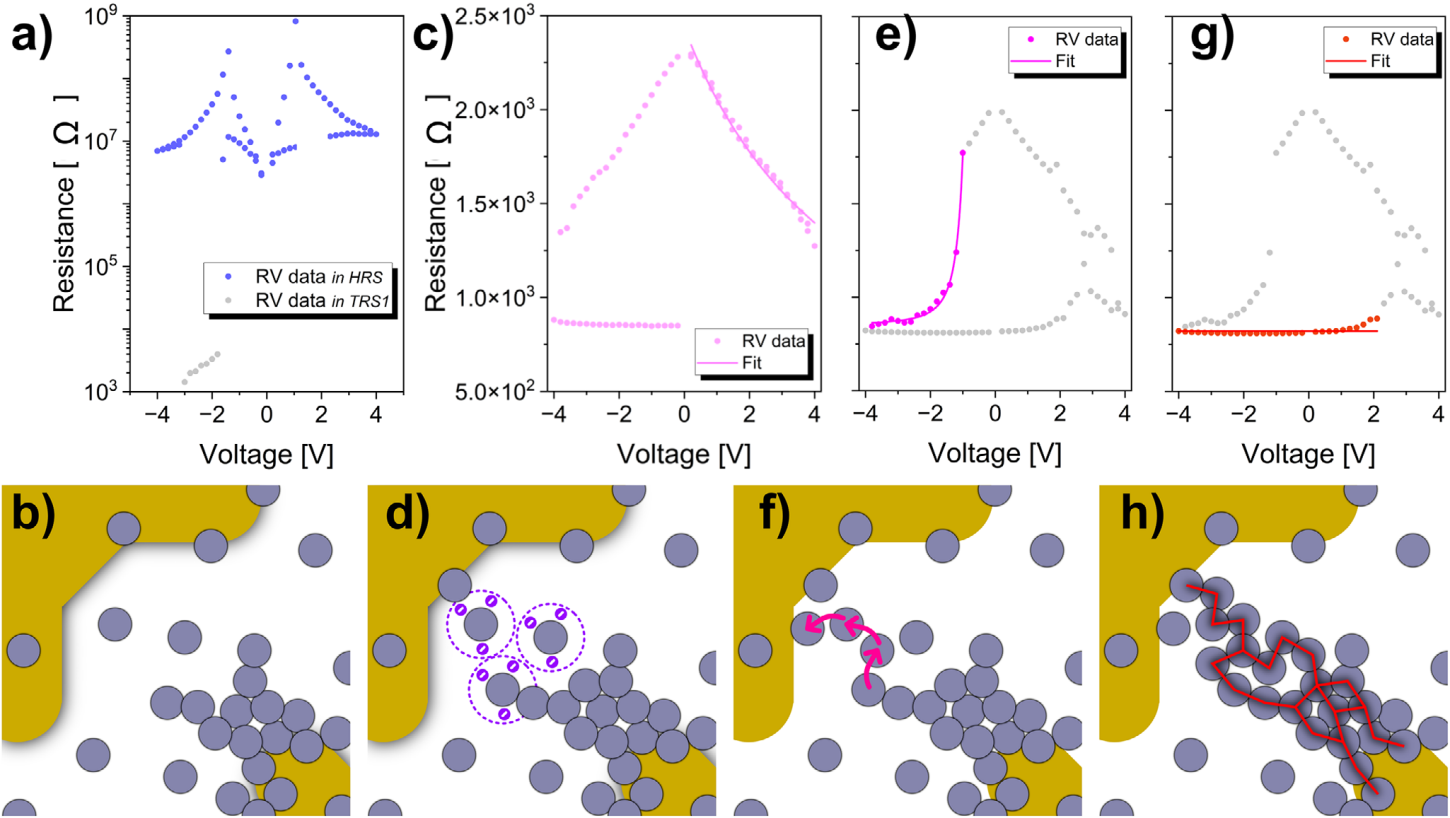
Examples of *R–V* switching characteristics and schematic representation of: a), b) HRS with large gaps; c), d) TRS1 with space‐charge limited conduction; e), f) TRS2 with tunneling conduction; g), h) LRS with Ohmic conduction. Lines on the *R–V* curves fit the data. Complementary *R–V* characteristics for all three types of nanofluids are available in the Supporting Information (see Figure S10, Supporting Information). *R–V* switching characteristics in Figure 4c), e), and g) have the same range in the Resistance axis.

The system is in the HRS when the resistance remains at a level comparable to that of the host PEG (see **Figure** **4**a). During this state, there are significant gaps larger than the size of NPs within the bridge, as schematically shown in Figure 4b. The intrinsic conductivity of PEG is due to dissociated water ions (≈3 wt.% of H_2_O absorbed by PEG^[^ ^27^ ^]^) or trace impurities related to synthesis or storage.^[^ ^36^ ^]^ During the *I*–*V* cycling, the HRS may stochastically switch either to the TRS or LRS.

*TRS*

The resistance in the TRS is lower than that in the HRS, but higher than in the LRS. This indicates that the network is in the unpercolated state, with the gaps within the bridge comparable to or smaller than the NP size. We have also observed that two types of *R‐V* dependencies can describe the TRS, which we will refer to as TRS1 and TRS2.

The TRS1 follows the dependence *R*∝*V^−1^* (Figure 4c), and we associate it with space‐charge‐limited conduction, schematically depicted in Figure 4d. In this regime, charge injection is limited by the accumulation of space charges rather than by the availability of free charge carriers. The current density is commonly determined by the Mott‐Garnet law $J=\frac{9}{8}\;\mu \varepsilon \varepsilon_{0}\frac{V^{2}}{L^{3}}$, where *J* – current density, *µ* – charge carrier mobility, *ε* – dielectric permittivity of the material, *ε_0_* – permittivity of vacuum, and *L* – thickness of the material.^[^ ^37^ ^]^ Here, the distance between the aggregates is relatively large but does not substantially exceed the NP size, so that the electrical double layers around the NPs overlap. In addition to conduction via electrons, the charge transport can also occur through dissociated water and dissolved Ag ions.

In TRS2, a stronger *R‐V* non‐linearity is observed, which can be best fitted by *R∝ V^−1^exp(V^−1^)* (Figure 4e), corresponding to the field‐driven Fowler‐Nordheim tunneling mechanism $I=A_{eff}\;\frac{e^{3}m}{8\pi hn\emptyset_{B}}E^{2}\exp\left(-\frac{8\pi \sqrt{2m}}{3he}\frac{{\emptyset_{B}}^{\frac{3}{2}}}{E}\right)$. Here, *A_eff_* – effective contact area, *h* – the Planck constant, *∅_B_* – barrier height, *E* – applied electric field, and *m* – the effective mass^[^ ^38^ ^]^ (additional details of fitting are given in Figure S13, Supporting Information). It is worth noting that the same mechanism is responsible for the conduction of NP‐based solid‐state networks, poised at the threshold of percolation with tunneling, that were successfully used as neuromorphic platforms for reservoir computing.^[^ ^39^ ^]^ In this case, the gaps are in the nanometer range, so that the resistance decays faster to the values close to the LRS. Nevertheless, the transition from the TRS2 to LRS and vice versa is gradual, which points to the existence of energy barriers that hinder an abrupt switching to direct metal‐metal contact between the NPs. Electrons can tunnel through these narrow gaps, as shown schematically in Figure 4f.

*LRS*

The LRS is formed when at least one path with ohmic conduction is created between the electrodes, as illustrated in Figure 4g,h. The switching to or from the LRS can occur through the formation or disruption of continuous paths of NPs that are in direct contact with each other.

The process of the resistive switching between individual resistance states can be followed based on the *I–V* pinched hysteresis and corresponding R‐V characteristics demonstrated in Figure S11 (Supporting Information). Initially, the system is in the LRS with linear *I–V* dependence. At a positive polarity of the biased electrode, the NPs are attracted to the positive potential, and at least one gap (or several gaps) appears, disrupting the direct connection between electrodes. Initially, the gap size is narrow, allowing tunnelling and TRS 1. If the gap size continues increasing, the tunnelling becomes impossible. The electrical current is due to the space charges, and TRS 2 is observed. When the gap's size overcomes the critical value (bigger than the NP size), the current abruptly decreases to values corresponding to the base PEG (HRS). Conversely, at a negative polarity of the biased electrode, NPs migrate electrophoretically toward the grounded electrode, decreasing the gap (or gaps) size and forming a long‐range conductive NP bridge. The current abruptly increases from the PEG level to the level of TRS when the *I*–*V* characteristic is non‐linear. When the direct metallic connection between the electrodes is repaired, the TRS switches to LRS with a linear *I*–*V* characteristic.

In addition to this electrophoretic rearrangement, an alternative pathway for resistive switching at nanoscale inter‐particle gaps traces back to electrochemical metallization. Here, similar to solid state memristive devices with diffusive switching characteristics, metal filament formation could be induced by Ag^+^ ionization, ion transport mediated by electric field, and reduction and nucleation. In contrast, filament dissolution could be governed by breakdown due to Joule heating, diffusion and surface energy reduction. Nevertheless, the validity of this mechanism in nanofluidic systems should be experimentally verified using different NPs, such as gold NPs.

### 2.3 Resistive Switching Dynamics Versus Molecular Mobility of PEG

**Figure 5:**
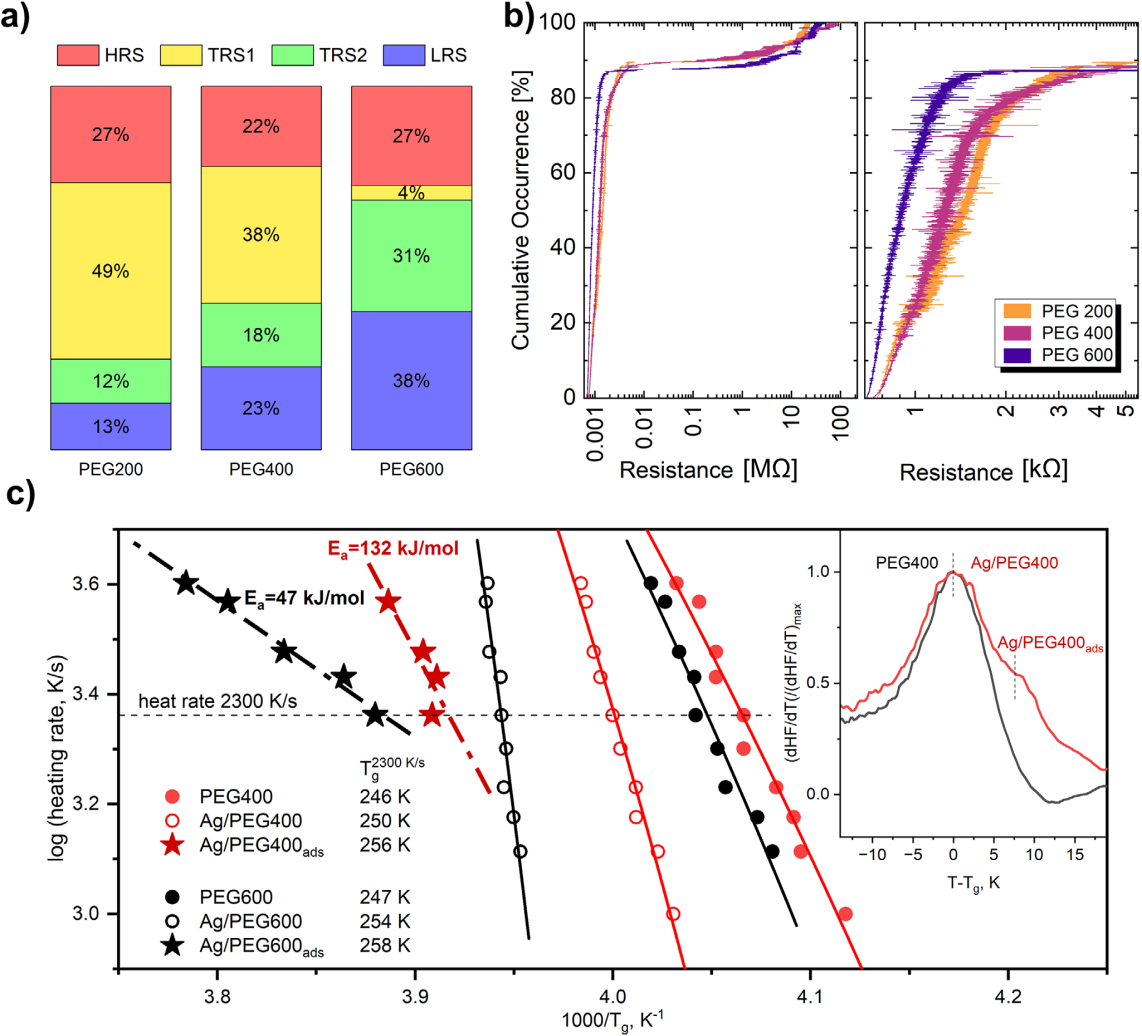
a) The relative occurrence of individual resistance states in Ag/PEG200, Ag/PEG400, and Ag/PEG600 nanofluids; b) cumulative occurrence plot of resistance levels for 200 *I–V* hysteresis cycles for Ag/PEG200, Ag/PEG400, and Ag/PEG600 nanofluids. To obtain the cumulative occurrence plot for each nanofluid, the observed resistance levels from the four subsections of each of the 200 *I–V* hysteresis cycles were sorted in ascending order. Cumulative occurrence represents the probability of observing a resistance level equal to or smaller than the respective value. Relaxation maps c) as log(heating rate) versus inverse of *T_g_*: filled and open circles indicate the segmental dynamics of PEG and Ag/PEG; asterisks indicate processes in the adsorbed layer; solid lines are fits of the VFT equation to the data; dashed lines are fits of the Arrhenius equation to the data. The inset shows the derivative of the heat flow of PEG400 (black line) and Ag/PEG400 (red line) versus reduced temperature for a heating rate of 4000 K s^−1^.

All nanofluids were studied regarding their resistive switching characteristics in 200 consecutive switching cycles by recording IV measurements, and cycling stability toward 200 consecutive cycles was observed. **Figure** **5**a shows the distribution of the relative occurrence of all resistance states on the *R‐V* characteristics. Figure 5b depicts the cumulative plot of the relative occurrence of the individual resistance levels for 200 hysteresis cycles of Ag/PEG200 (orange line), Ag/PEG400 (red line), and Ag/PEG600 (blue line) nanofluids (details of the plotting protocol can be found in the Supporting Information). The HRS occurrence ranges from 22% to 27% for all PEG types. The general trend can be observed in the probability of the LRS, which increases with the molecular mass of PEG. A distinct trend was also observed for TRS 1 and TRS 2, where the occurrence of TRS1 decreases, and TRS2 increases with molecular mass.

In the context of liquid resistive switching, the uniqueness of the Ag/PEG nanofluid lies in the use of polymer as a host medium, with macromolecular dynamics providing an additional level of complexity to the system. To correlate the observed switching dynamics with the molecular mobility of PEG, we performed measurements of the glass transition temperature *T_g_* at different heating rates for PEGs and Ag/PEGs using flash scanning calorimetry (FSC). The Ag/PEG pastes were prepared with a concentration of 20 mg mL^−1^ by centrifugation of Ag/PEG nanofluids to simulate the conditions of NPs in the conductive bridge. The inset of Figure 5c shows an example of the measurements presented as the first derivative of the heat flow with respect to temperature (*dHF/dT*) for PEG400 and Ag/PEG400. For pure PEG, only one peak is observed in the derivative, which corresponds to the glass transition of the polymer related to the segmental dynamics. For Ag/PEG400, the derivative of the heat flow is bimodal with a second process at higher temperatures. Therefore, two Gaussians were fitted to the data. We attribute the additional process with the higher *T_g_* to a PEG layer adsorbed on the surface of Ag NPs or agglomerates, where the adsorption leads to significantly restricted segmental dynamics.^[^ ^40^ ^]^

Figure 5c shows the PEG relaxation maps built by plotting the logarithm of the heating rate versus the inverse *T_g_* for PEG400 and PEG600 with or without Ag NPs (the *T_g_* of PEG200 could not be measured due to the limitations of the Flash DSC1). As expected, the PEGs show a *T_g_* in the sub‐zero Celsius temperature range, confirming the melt state at room temperature. The slight increase in *T_g_* with molecular mass is consistent with a slowing down of segmental mobility. The temperature dependence of the heating rate can be described by the Vogel/Fulcher/Tammann (VFT) equation, as expected for a glass transition (solid lines in Figure 5c).^[^ ^41^, ^42^, ^43^ ^]^ The Ag/PEG samples show a higher *T_g_* of the PEG matrix compared to their host liquids (the reference values of *T_g_^2300 K/s^* are given in the legend of Figure 5c), which indicates that NPs strongly restrain the mobility and relaxation behavior of polymer segments. As the molecular mobility of the matrix is slower (*T_g_* higher) compared to the unfilled PEG, the influence of the slowed‐down molecular mobility of the adsorbed layer percolates through the entire matrix of the nanocomposites. Interestingly, we observed similar bimodal *T_g_* in Cu/polyether thin films in a previous study.^[^ ^44^ ^]^ It is worth mentioning that the difference between the temperature dependence of Ag/PEG400 and Ag/PEG600 is larger than that for the unfilled samples.

Another interesting aspect emerges from the analysis of the temperature dependence of the heating rates for the adsorbed layer, which can be approximated by an Arrhenius equation characterized by an apparent activation energy *E_a_* (dashed lines in Figure 5c). The Arrhenius fit gives a value of the activation energy of 132 kJ mol^−1^ for the molecular fluctuations in adsorbed Ag/PEG400. This is much higher than the activation energy of 47 kJ mol^−1^ found in the adsorbed Ag/PEG600, indicating that there are higher energy barriers to segmental fluctuations in the adsorbed Ag/PEG400, suggesting a denser adsorbed layer. In this case, the adsorbed layer consists mainly of train conformations (polymer segments in direct contact and strong adsorption to the surface), while for Ag/PEG600, a part of the adsorbed layer contains more loops and dangling ends (few segments of the polymer chain are pinned to the substrate, whereas the majority of the chain is extended away from the substrate) besides the dense adsorbed layer. These loops and ends will allow for a higher molecular mobility in the adsorbed layer for Ag/PEG600 compared to that of Ag/PEG400.

The same conclusion of the denser adsorbed layer in lower molecular mass Ag/PEGs can be drawn based on the De Gennes formalism^[^ ^45^ ^]^ that relates the thickness of the adsorbed layer *L* to the grafting density *σ* (the ratio of the attached chains to the mobile chains that do not adhere to the surface). The thickness is given by the relationship *L = naσ^1/2^*, where *n* is the number of monomers per polymer chain and *a* is the length of one monomer. The previous investigation found that PEG segments adsorbed onto NPs in a low grafting density regime, with the thickness of the adsorbed layer very close to the characteristic size of an ideal chain *R_0_*.^[^ ^27^ ^]^ Considering that in polymer melts, *R_0_* = *an^1/2^*, where *a* is the length of one monomer (a = 0.35 nm for PEG,^[^ ^46^ ^]^), and assuming that *L* is approximately equal to *R_0_*, we can conclude that *σ* decreases with increasing molecular mass of PEG (Table S3, Supporting Information). This means the contribution from the attached PEG segments decreases for the PEGs with higher molecular weights, leading to a higher probability of metal‐metal contact (higher probability of the LRS) in PEG600.

The same line of argumentation also explains why the *ξ*‐potential of Ag NPs decreases with the molecular mass of PEG (Table 1). The growth of the adsorption of the polymer layer on the NPs follows a two‐step process.^[^ ^40^ ^]^ For short times, the adsorption follows a linear time dependence, where individual segments attach directly to the NPs surface at the cost of energy, creating many interfacial contacts. After a certain period, the linear relationship changes to a logarithmic one, where the adsorbed layer continues to grow further by the diffusion of segments through the already existing adsorbed layer at the cost of entropy. Due to the lower entropic barriers in PEG200 compared to PEG600, this stage is “easier” for the former, resulting in a denser adsorbed layer. As mentioned above, DFT calculations revealed that a negative charge is formed on the PEG molecule and that the higher number of surface contacts leads to a higher charge.^[^ ^27^ ^]^ This is because a denser packing of chains in the adsorbed layer increases the *ξ*‐potential for PEG200 compared to PEG600.

It is also interesting to note that when the system is in the TRS, the probability of TRS1 decreases, and the probability of TRS2 increases as the molecular mass of PEG increases. This means that, in the case of PEG600, NPs preferentially stay closer to each other, separated by only a thin adsorbed layer (≈1 nm). In contrast, in PEG200, NPs preferentially have greater distances from each other, corresponding to the length scale of the NP size (tens of nanometers). We also correlate this phenomenon with the *ξ*‐potential developing on Ag NPs. A higher *ξ*‐potential in PEG200 creates stronger Coulomb repulsion between neighboring NPs and causes them to stay apart. Conversely, a lower *ξ*‐potential in PEG600 results in lower electrostatic barriers and weaker colloidal stability of this system. Moreover, considering the structure of the adsorbed layer, it could be understood why TRSs for Ag/PEG200 are mainly due to space charge conduction while those of Ag/PEG600 are dominated by tunneling (see Figure 5a). Interestingly, in the nanofluids, the interaction between the nanoparticles and the polymer host fluid adds further pathways for reconfiguration of resistance states beyond the formation and dissolution of atomic filaments via ionization, transport, and nucleation. Such filamentary memristive switching, which relates to the release of Ag^+^ ions, their migration and transport guided by an electric field, and their reduction and nucleation back to the metallic state, is commonly reported, for example, for electrochemical metallization memories in solid‐state devices.^[^ ^47^ ^]^ In Ag‐based nanofluids, electrophoretic rearrangement presents a parallel pathway to achieve reconfigurability in the resistance states. While both mechanisms are governed by transport in the presence of electric fields, based on the presented data, we hypothesize that electrochemical formation and dissolution of metal filaments bridging the dielectric gaps between NPs is not dominant in melted polymer matrices, although it requires further experimental verification.

## 3 Conclusion

The Ag/PEG nanofluids were prepared by sputter‐driven synthesis of 56‐nm‐sized Ag NPs and their subsequent loading into PEG melts of different molecular masses. When in PEG, NPs bear a negative ξ‐potential and can be manipulated by an electrical field, resulting in electrophoretic transport along the electric field between the electrodes. This study showcases the initialization of long‐range NP bridges in a planar two‐terminal configuration and proposes a corresponding equivalent circuit, reports on the diversity of reconfigurable resistance states, and investigates the role of the host liquid on resistive switching in Ag/PEG nanofluids.

### 3.1 Initialization of Long‐Range NP Bridges

Nanofluids are initialized for resistive switching by applying a constant DC bias between two electrodes. In this way, NPs from the nanofluidic reservoirs can be attracted toward the positively biased electrode and brought to the vicinity of the interelectrode gap. An equivalent circuit model involving Ag NPs, PEG matrix, and the substrate with the electrode array was proposed to represent the main electrical components of the nanofluidic system. *I‐t* measurements during the initialization step and complementary impedance spectroscopy were employed to determine the capacitive and resistive components of the model. The initialization step at constant bias resembles similarities to the electroforming in solid‐state filamentary memristive devices, posing the challenge of a transition toward forming‐free nanofluids for future developments in nanofluid resistive switching. However, in contrast to the electroforming in solid‐state devices, the long‐range mobility of NPs and NP agglomerates in nanofluids promises the potential for directed growth of long‐range connections between multiple terminals for tailored connection schemes (potentially beyond two dimensions).

### 3.2 Reconfigurability of Resistance States in Long‐Range Conductive NP Bridges

After initialization, cycling *I–V* measurements were performed to reveal a polarity‐dependent resistive switching in the long‐range conductive NP bridge. The SET voltage was associated with the negative polarity of the biased electrode, which resulted in enriching the inter‐electrode gap with NPs. The RESET voltage was related to the positive polarity of the biased electrode, which led to the depletion of NPs in the inter‐electrode gap. This study showcases the diverse range of reconfigurable resistance states in long‐range conductive NP bridges in Ag‐based nanofluids:

1. A low resistance state, which corresponds to the presence of a percolation path at the long‐range conductive NP bridge.
2. A high resistance state, which corresponds to the dissolution of the percolation path and filamentary connections, resulting in discontinuity in the long‐range NP bridge.
3. Intermediate transition resistance states, which correspond to nanoscale gaps along the long‐range conductive NP bridge, resulting in non‐linear *I–V* characteristics. In TRS1, agglomerates of NPs are separated by distances comparable with the NP size, the electrical double layers surrounding the NPs overlap, and the conductivity is space charge‐limited, obeying the dependence *R*∝*V^−1^*. In TRS2, the agglomerate gaps are in the nanometer range, and the conductivity is given by electron tunneling, obeying the dependence *R∝ V^−1^exp(V^−1^)*.

This novel report on additional transition resistance states with non‐linear *I–V* characteristics expands the exploration space for the design of metal‐dielectric nanofluids for mimicking axonal growth and synaptic plasticity in networks with growable connections.

### 3.3 The Role of the Host Liquid on Resistive Switching in Ag/PEG Nanofluids

The occurrence of the individual resistance states was found to be governed by the molecular mobility of PEG, which was assessed by FSC. Depending on the molecular mass, PEG molecules adsorb on the surface of Ag NPs, forming adsorbed layers of different densities. Smaller molecules of PEG 200 have higher mobility and adsorb preferentially in train configurations with multiple surface contacts, creating a denser adsorbed layer and acquiring a larger negative potential. Consequently, the agglomerates of NPs are hindered from going into direct metal‐metal contact, and the bridge remains preferentially in TRS1. Larger molecules of PEG600 have lower mobility, adsorbing with loops and dangling ends that provide fewer direct surface contacts. Thus, the Ag/PEG600 sample has a less dense adsorbed layer and acquires a smaller negative potential, allowing the agglomerates to come into direct metal‐metal contact (the LRS) or remain at smaller distances from each other (the TRS2) more frequently. Consequently, our findings suggest that the choice of the polymer offers a pathway to diversify the resistive switching dynamics in electrophoretically assembled NP bridges in nanofluidic systems.

## 4 Experimental Section

### Materials

Ag sputtering targets (3 inches, 3 mm thick, 99.99% purity) were procured from SAFINA, a.s. (Czech Republic). Liquid PEGs with average molecular weights of *M_n_* = 200, 400, and 600 g/mol were supplied by Merck Life Science spol. s r.o. (Czech Republic). All PEGs were filtered through 200 nm polystyrene filters to minimize impurities and remove dust and any polymerized particles prior to the preparation of the nanofluids.

### Fabrication of Ag/PEG Nanofluids

Ag/PEG nanofluids were synthesized using a one‐step, surfactant‐free magnetron sputtering technique combined with gas‐phase aggregation. The metal vapor condenses in cold argon to form NPs, which were then transported directly into the PEG melt.^[^ ^25^ ^]^ This method has proven effective for producing Cu/PEG,^[^ ^48^ ^]^ Au/PEG,^[^ ^49^ ^]^ Cu/Ag/PEG,^[^ ^27^ ^]^ and various transition metal nitride‐based nanofluids.^[^ ^50^, ^51^, ^52^ ^]^ The average size of Ag NPs was 56 ± 16 nm, and the deposition time was adjusted to yield a nanofluid concentration of 2000 µg mL^−1^. To ensure homogeneity, each nanofluid was sonicated for 1 min using an ultrasonic homogenizer (Fisherbrand Model 50, Fisher Scientific, U.S.A.).

### Resistive Switching Characterization

Electrical measurements were conducted to investigate resistive switching in Ag/PEG nanofluids deposited onto Pt electrode arrays on Si wafers. The fabrication process for electrodes was previously described in ref. [25]. A novel arrangement was designed with two coaxially aligned active electrodes located at the center of the substrate, one biased and the other grounded. The schematic is shown in Figure S1 (Supporting Information). A nanofluid droplet of 0.3 µl was placed over the tips of the electrodes. *I–t* measurements at constant voltage and *I–V* sweeps were performed using a commercial probe station (BD‐6, Everbeing Inc., Taiwan) and a source meter (Keithley 2400, Tektronix, U.S.A.) connected to Pt electrodes through tungsten needle contacts. To avoid system damage due to excessive current, the compliance was set at 0.01 A. Data acquisition was performed at a temporal resolution of ≈0.1 s for *I–t* measurements. For *I–V* sweeps, a staircase voltage ramp was used with up to 192 steps per sweep. The accumulation of NPs within the interelectrode gap was monitored in situ using an optical microscope (PSM‐1000, Motic, Hong Kong) with image capture via a CCD camera (Moticam 3+, Motic, Hong Kong). The morphology and chemical composition of NP agglomerates were analyzed using a SEM (JSM‐7200F, Jeol Ltd., Japan) equipped with an EDX detector after excess PEG was removed by rinsing with isopropanol.

### ξ‐Potential and Electrophoretic Mobility Measurement

The *ξ*‐potential and electrophoretic mobility of Ag NPs in different PEGs were studied using Light Scattering Electrophoresis (Zetasizer NanoZS ZEN3600, Malvern Panalytical, United Kingdom). Measurements were performed on nanofluids with a concentration of 50 µg mL^−1^ to prevent strong light absorption. No dilution or filtration of the samples was carried out. A universal dip cell with closely spaced parallel palladium electrodes was used due to the lower dielectric constant and higher viscosity of PEG as compared to aqueous solutions.^[^ ^35^, ^53^, ^54^ ^]^ The number of automatic runs was limited to 60, and the *ξ*‐potential was calculated using the Hückel approximation.^[^ ^27^ ^]^

### Fast Scanning Calorimetry

FSC was carried out by a power‐compensated Flash DSC 1 (Metter Toledo, Switzerland).^[^ ^55^ ^]^ FSC was based on adiabatic chip calorimetry, which allows for heating rates from 10 K/s to 10^4^ K/s due to the micro‐designed UFS2 chip sensor. The cooling rates were one order of magnitude lower. The employed sample masses were in the range from 200 to 500 ng. The base temperature of the sensors was controlled by a Huber TC 100 intercooler. To avoid crystallization of PEG, the samples were cooled down to ‐90 °C at a 10^3^ K s^−1^ rate prior to the heating run, which was carried out at different heating rates. To estimate the glass transition temperature of PEG, a sigmoidal function was fitted to the heat flow in the range of the glass transition. This function was differentiated with respect to temperature, which resulted in a peak in the derivative. The peak was fitted by a Gaussian function, where the maximum was taken as the glass transition temperature *T_g_*. For the Ag/PEG samples, two Gaussians were fitted to the derivative of the sigmoidal function, as discussed in the text below.

### Simulation of the Electric Field

The electrostatic field was simulated using the Siemens STAR‐CCM+ R8 Multiphysics software with double precision, focusing on a 3D model. The simulation involved an electrode biased at a potential of +4 V, while other electrodes were grounded. PEG with a permittivity of 14 F m^−1^ was used as a medium. Assuming that the electric field was constant with time, time‐dependent terms were neglected in Maxwell's equations. A polyhedral mesh was employed to capture the 3D geometry of the system. Thermal effects were excluded from the analysis.

## Conflict of Interest

The authors declare no conflict of interest.

## Supporting information



Supporting Information

Supplemental Video 1

Supplemental Video 2

## Data Availability

The data that support the findings of this study are available from the corresponding author upon reasonable request.
