# Supplementary material for: Unveiling the Fundamental Principles of Reconfigurable Resistance States in Silver/Poly(Ethylene Glycol) Nanofluids
Source: Adv Sci (Weinh). 2025 Jun 26;12(35):e05103. doi: 10.1002/advs.202505103 (PMC12462977; doi:10.1002/advs.202505103)
Supplement: Supplementary file 1 — Supporting Information [file ADVS-12-e05103-s002.docx]

Supporting Information

Unveiling the Fundamental Principles of Reconfigurable Resistance States in Silver/Poly(ethylene glycol) Nanofluids

Daniil Nikitin*, Kateryna Biliak, Mariia Protsak, Blessing Adejube, Suren Ali-Ogly, Kateřina Škorvanková, Veronika Červenková, Ronaldo Katuta, Marco Tosca, Jan Hanuš, Zulfiya Černochová, Peter Černoch, Petr Štěpánek, Oleksandr Boiko, Paulina Szymoniak, Andreas Schönhals, Franz Faupel, Hynek Biederman, Alexander Vahl*, and Andrei Choukourov

*Corresponding author. E-mail: [daniil_nikitin@kmf.troja.mff.cuni.cz](mailto:daniil_nikitin@kmf.troja.mff.cuni.cz), [alva@tf.uni-kiel.de](mailto:alva@tf.uni-kiel.de)

**SI 1.** **Initializing the bridge in different PEGs**

The chip used for electrical measurements is schematically illustrated in **Figure SI 1**. It consists of a 10 × 10 mm Si wafer with a 3 µm-thick surface oxide layer grown via thermal annealing. Planar Pt electrode arrays were created using photolithography in a cleanroom environment. Electrodes 1 and 7 were used in the current work for the initialization of conductive bridge formation and resistive switching measurements. They were connected to a Keithley 2400 source meter (Tektronix, U.S.A.) via needle-shaped tungsten contacts from the commercial probe station BD-6 (Everbeing Inc., Taiwan). Electrode 1 was biased, while electrode 7 was grounded. Two independent groups of electrodes 2 – 6 and 8 – 12 were planned to be utilized for multi-electrode measurements. In the current work, they were not used in the measurements. Electrodes 2, 4, 6, 8, 10 and 12 were left under floating potential, whereas electrodes 3, 5, 9 and 11 were grounded. Considering their distance from the active electrodes, they do not influence the electrical measurements performed between electrodes 1 and 7. A 0.3 µL droplet of nanofluid was placed in the center of the chip using a micropipette.


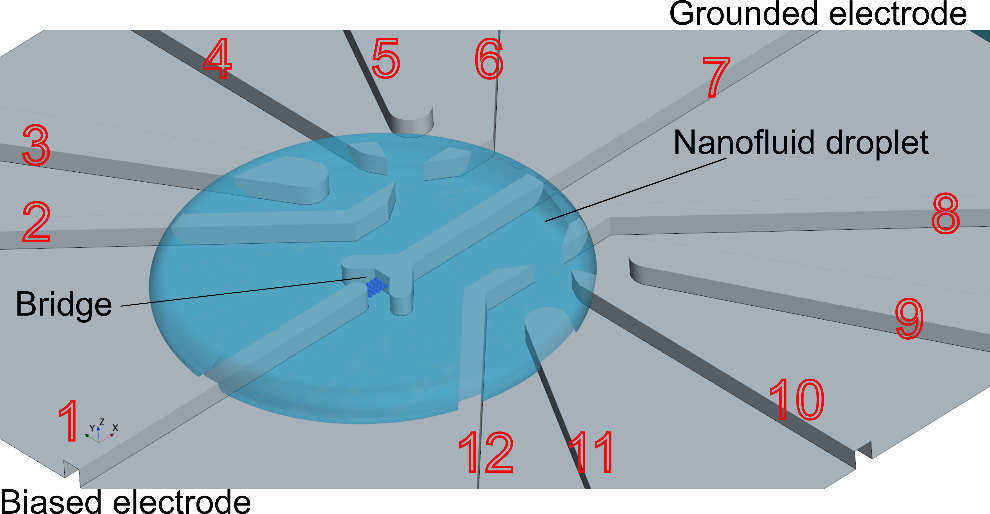


**Figure SI 1** Scheme of the electrical measurement on the Ag/PEG nanofluid

Two types of electrical characteristics were acquired during the experiment.

(1) *I–t* characteristics were measured with a constant bias voltage V_bias_ = +4 V to form a conductive pathway by inducing the movement of Ag nanoparticles (NPs) towards the biased electrode through electrophoresis and their aggregation within the interelectrode gap.

(2) Cycling *I–V* characteristics were obtained to evaluate the resistive switching behaviour of the pre-formed conductive path.

In the pristine state, before any electrical stimulation, the Ag NPs and NP agglomerates are evenly distributed across the whole nanofluid drop volume. During the initialization step, NPs are attracted towards the positively biased electrode, and consequently, the distribution of NPs and NP agglomerates in the inter-electrode gap is changed. During this process, the nanofluid arrangement in the interelectrode gap is brought into a state where the subsequent application of sawtooth voltage pulses (IV hysteresis measurements) can induce reconfigurable transitions between low resistance state (LRS), transition resistance states (TRS) and high resistance state (HRS) in the long-range conductive NP bridge. In the pristine state, the electrical characteristics of a nanofluid are, to a large extent, dominated by the base conductivity of the chosen host liquid, in this case PEG200, PEG 400 and PEG 600. During initialization, we observe first the decay in current response due to the intrinsic capacitive components, followed by gradual changes in the current response. We describe initialization as complete once the first spikes in the current response occur. Although the nanofluid at this stage is still in its HRS, and these spikes are orders of magnitude below the current response in the LRS or TRS, the occurrence of the spikes is a clear deviation from the behaviour of a host liquid. Thus, the accumulation of NPs and NP agglomerates in the vicinity of the inter-electrode gap has initialized the nanofluid system into a state where minor changes in the distance between neighbouring NPs in the long-range NP bridge can induce reconfigurable changes between HRS, TRS and LRS.

**Video SI 1** illustrates the movement of Ag NPs in a nanofluid under the influence of an applied electric field. When a bias voltage is applied, the NPs immediately begin to move, accumulating near the biased electrode and in the interelectrode space. As the concentration of NPs goes beyond a certain threshold, current bursts appear on the *I–t* characteristic.

**Video SI 1** The exemplary video demonstrates the formation of a conductive bridge between the electrodes in Ag/PEG400 nanofluid. The V_bias_ = +4 V was applied to the biased electrode.

**SI 2. SEM-EDX characterization of the conductive path**

Scanning electron microscopy and energy-dispersive X-ray analysis (SEM-EDX) was performed on the conductive path formed within the interelectrode gap after removing excess PEG by rinsing with isopropanol and drying in a stream of nitrogen. The SEM images in **Figures SI 2a–c** reveal that PEG removal was incomplete, as a polymer shell remains surrounding both the bulk of the conductive path and individual NPs (see **Figure SI 2c**). The EDX map of carbon distribution (**Figure SI 2d,e**) confirms this observation, showing an elevated concentration of elemental carbon that coincides with the NP-based bridge connecting the electrodes. Additionally, the EDX map of Si distribution (**Figure SI 2f**) closely matches the elemental oxygen distribution in Figure 1d, indicating that the observed oxygen is primarily associated with the surface oxide layer of the chip.


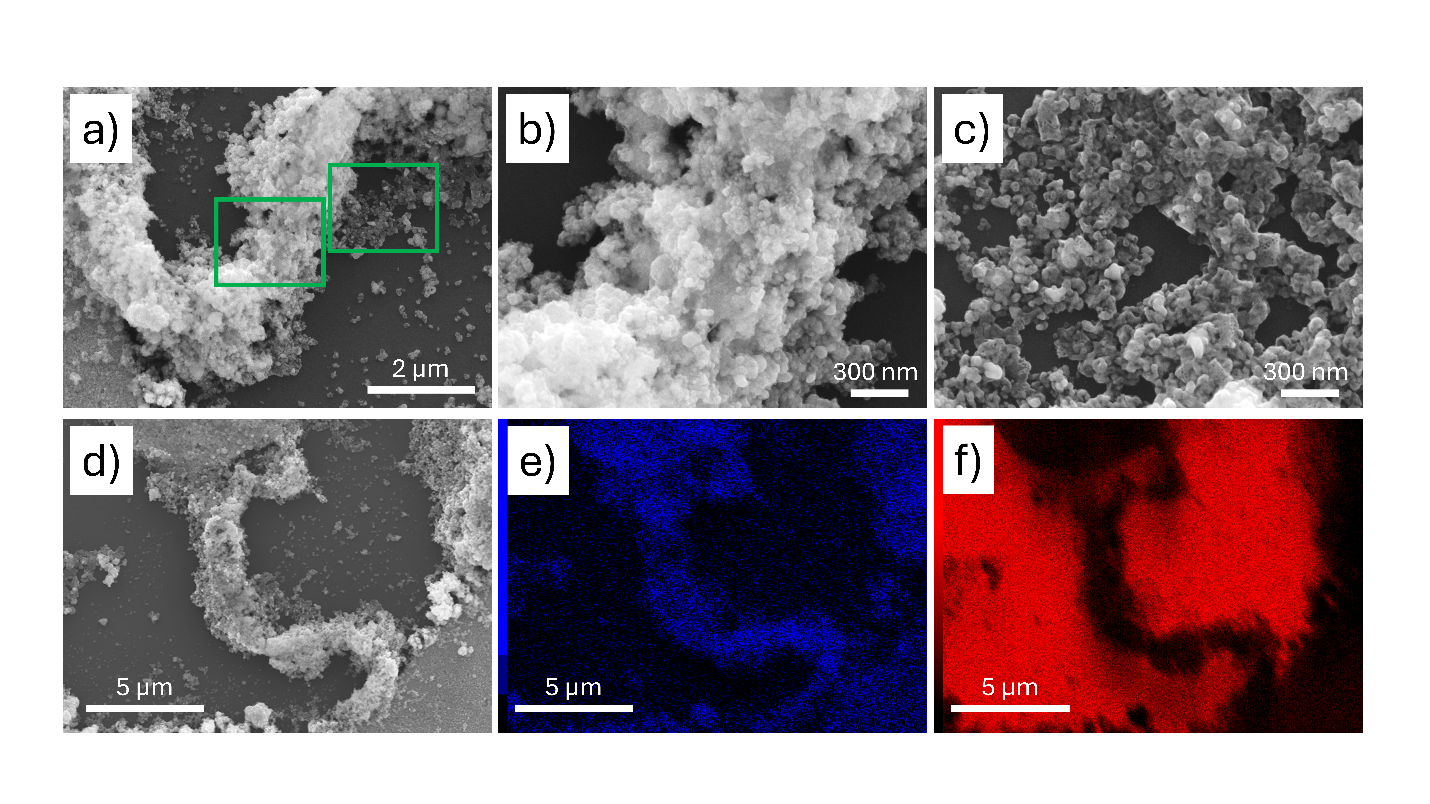


**Figure SI 2 a** The SEM image of the conductive bridge formed between the Pt electrodes in Ag/PEG400 nanofluid at the applied voltage of +4 V. The green frames correspond to the regions of interest represented at higher magnification in **b** and **c**. **d** The SEM image of the region used to acquire the EDX maps. **e** The distribution of carbon (blue) and **f** the distribution of silicon (red).

**SI 3. Fitting of *I–t* characteristics and electrophysical parameters of Ag/PEG nanofluids**

The *I–t* characteristics of Ag/PEG200 and Ag/PEG600 were fitted using a three-exponential function, similar to the *I–t* characteristics of Ag/PEG400 presented in the article:

$i=i_{0}+C_{1}\times\exp\left( -\frac{t}{\tau_{1}} \right)+C_{2}\times\exp\left( -\frac{t}{\tau_{2}} \right)+C_{3}\times\exp\left( -\frac{t}{\tau_{3}} \right)$ (1).

Exemplary *I–t* curves with corresponding fits for Ag/PEG200 and Ag/PEG600 are shown in **Figures SI 3a** and **SI 3b**, respectively. The capacitance components and time constants obtained from the fits are summarized in **Table 2** of the article. The three-exponential function provides the best fit for the experimental data, as confirmed by R-squared values exceeding 0.99.

Each exponential term corresponds to a specific capacitance component in the system. C_1_ and τ_1_ represent the capacitance of the gaps between the NPs distributed in the interelectrode gap. C_2_ and τ_2_ are associated with the capacitance between the Pt electrodes through the PEG. C_3_ and τ_3_ correspond to the capacitance between the electrodes via the surface oxide layer on the chip.

To validate this interpretation, *I–t* characteristics were also measured for control PEGs without NPs and fitted using a two-exponential model, excluding the C_1_ and τ_1_ components:

$i=i_{0}+C_{2}\times\exp\left( -\frac{t}{\tau_{2}} \right)+C_{3}\times\exp\left( -\frac{t}{\tau_{3}} \right)$ (2).

The fitted I–t characteristics for the control PEGs are shown in **Figure SI 4**. A two-exponential function provides a good fit for all three host liquids, yielding R-squared values close to or greater than 0.99. The electrophysical parameters (C_2_, τ_2_, C_3_, and τ_3_) presented in **Table SI 1** correlate with those determined for Ag/PEG nanofluids, with the exception of PEG400. This deviation in values may be due to variations in the water content of PEG and differences in the oxide layer thickness on the measurement chip. Nevertheless, the order of magnitude of PEG400 is still comparable to that of Ag/PEG400.

**Figure SI 3** Exemplary *I-t* characteristics with corresponding exponential fits for **a** Ag/PEG200 and **b** Ag/PEG600 nanofluids

**Figure SI 4** *I-t* characteristics of different PEGs without NPs fitted with the two-component exponential model. The electrophysical characteristics determined by fitting are presented in Table SI 1.

**Table SI 1** Fitting parameters determined from the exponential fits of I-t characteristics corresponding to the PEGs without NPs

| M_n_ of PEG (g/mol) |  | i_0_ (A) | C_2_ (nF) | τ_2_ (s) | C_3_ (nF) | τ_3_ (s) |
| --- | --- | --- | --- | --- | --- | --- |
| 200 |  | 2.3 ×10^-7^ | 130 | 15.3 | 260 | 253.0 |
| 400 |  | 7.2 ×10^-8^ | 57 | 2.6 | 41 | 111.0 |
| 600 |  | 1.1 ×10^-7^ | 58 | 11.0 | 51 | 310.5 |

**SI 4. Capacitance Measurements of PEG400 and Ag/PEG400 Nanofluid**

The capacitance of PEG400 and Ag/PEG400 nanofluids was measured experimentally using impedance spectroscopy. A custom-built measurement cell was designed for this purpose, consisting of a quartz glass cuvette equipped with parallel electrodes made from conductive copper tape. The schematic of the cell and its most important dimensions are shown in Figure SI 5. Capacitance measurements were carried out using a HIOKI LCR meter IM3536 (Hioki Europe GmbH, Germany) over the frequency range of 4 – 10^7^ Hz. The measurements were performed at room temperature and under ambient conditions.

Figure SI 5 presents the frequency-dependent capacitance of PEG400 and Ag/PEG400 nanofluids after accounting for the contribution of the surface capacitor that forms between the undisturbed contact areas of the electrodes. The trends observed are consistent with previous reports on PEG behaviour. Capacitance increases as the AC frequency decreases, reaching its highest values at the lowest frequencies. This behaviour is attributed to electrode polarization, where charge carriers accumulate at the liquid/electrode interface, forming electric double layers that increase capacitance ^[1]^. Additionally, the highly capacitive region at low frequencies may be due to ionic impurities, such as protons from absorbed water in PEG. At higher frequencies, long-range charge drift is suppressed, resulting in a stable capacitance value. Interestingly, Ag/PEG400 has a higher capacitance than pure PEG400, suggesting the contribution of nanoparticle-related effects. At 4 Hz, the measured capacitance values were around 10^-7^ F, which is in good agreement with the fitted C_2_ values obtained from the *I – t* characteristics (Table 2, Table SI 1). This suggests that the DC regime can be approximately described by the AC regime at zero frequency, confirming the validity of our fitting approach.


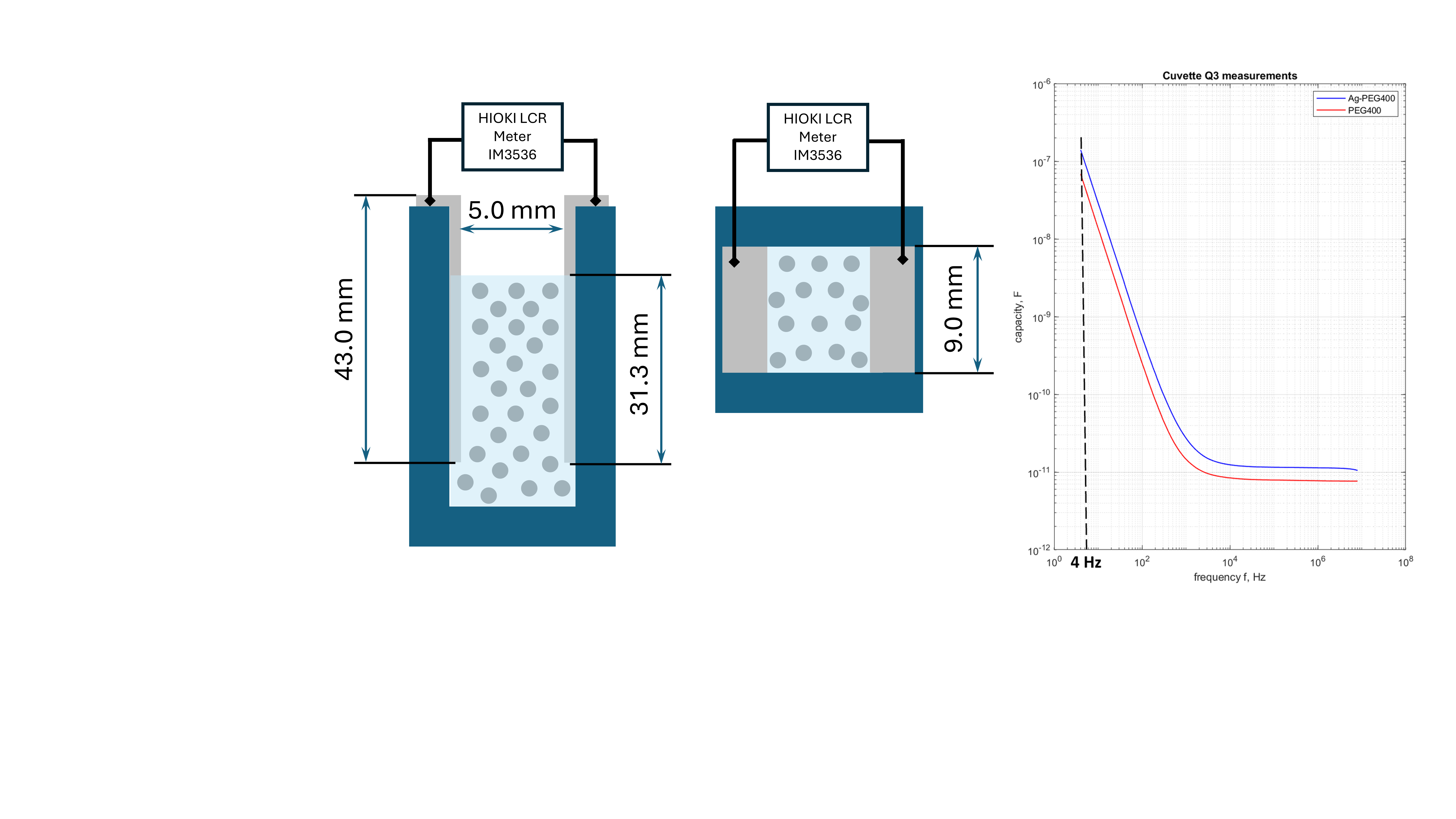


**Figure SI 5** The scheme of the liquid cell (side and top views) used for the measurement of capacitance by impedance spectroscopy. The graph illustrates the frequency-dependent capacitance of PEG400 and Ag/PEG400 nanofluid.

**SI 5. Influence of PEG’s viscosity on the kinetics of conductive path formation**

The physicochemical properties of the base fluid, in particular viscosity, play a significant role in the resistive switching kinetics. To quantify this effect, we measured the size of the aggregation zone near the biased electrode tip and at a distance of 20 µm from the tip. This was done by superposing a semitransparent electrode mask (see **Figure SI 6**) with four bars on optical microscopy images taken at different time points after applying a +4 V bias.


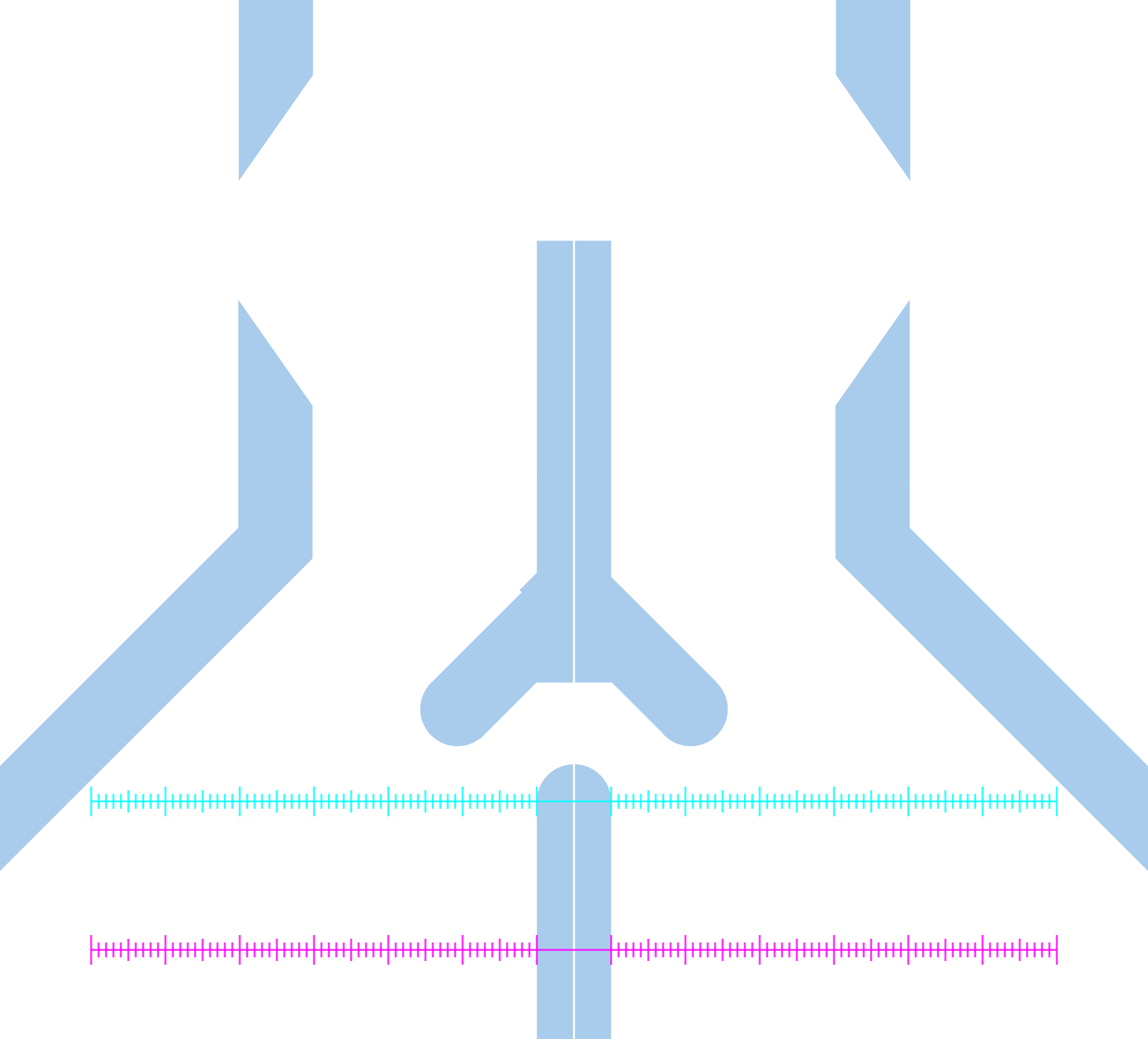


**Figure SI 6** The semi-transparent mask used for the evaluation of the mean aggregation length

**Figures SI 7a** and **b** show representative aggregation zones in Ag/PEG200 and Ag/PEG600 after 15 minutes, while **Figures 7c,d** illustrate the kinetic dependencies of aggregation length growth for each PEG type. The fastest agglomeration zone growth occurred in PEG200, which has the lowest viscosity (0.063 Pa·s), while slower growth was observed in the more viscous PEG400 and PEG600. Notably, the difference in growth rate is most pronounced near the tip, where the electric field strength is highest; at a distance of 20 µm, the growth rates converge due to the reduced field strength. Interestingly, in Ag/PEG200 and Ag/PEG400, the aggregation region near the tip was broader than 20 µm apart after 15 minutes, indicating that this time was generally sufficient for the bridge to overlap the interelectrode gap. In contrast, for Ag/PEG600, NP accumulation had only begun near the tip, with a slightly broader agglomeration zone farther from it.


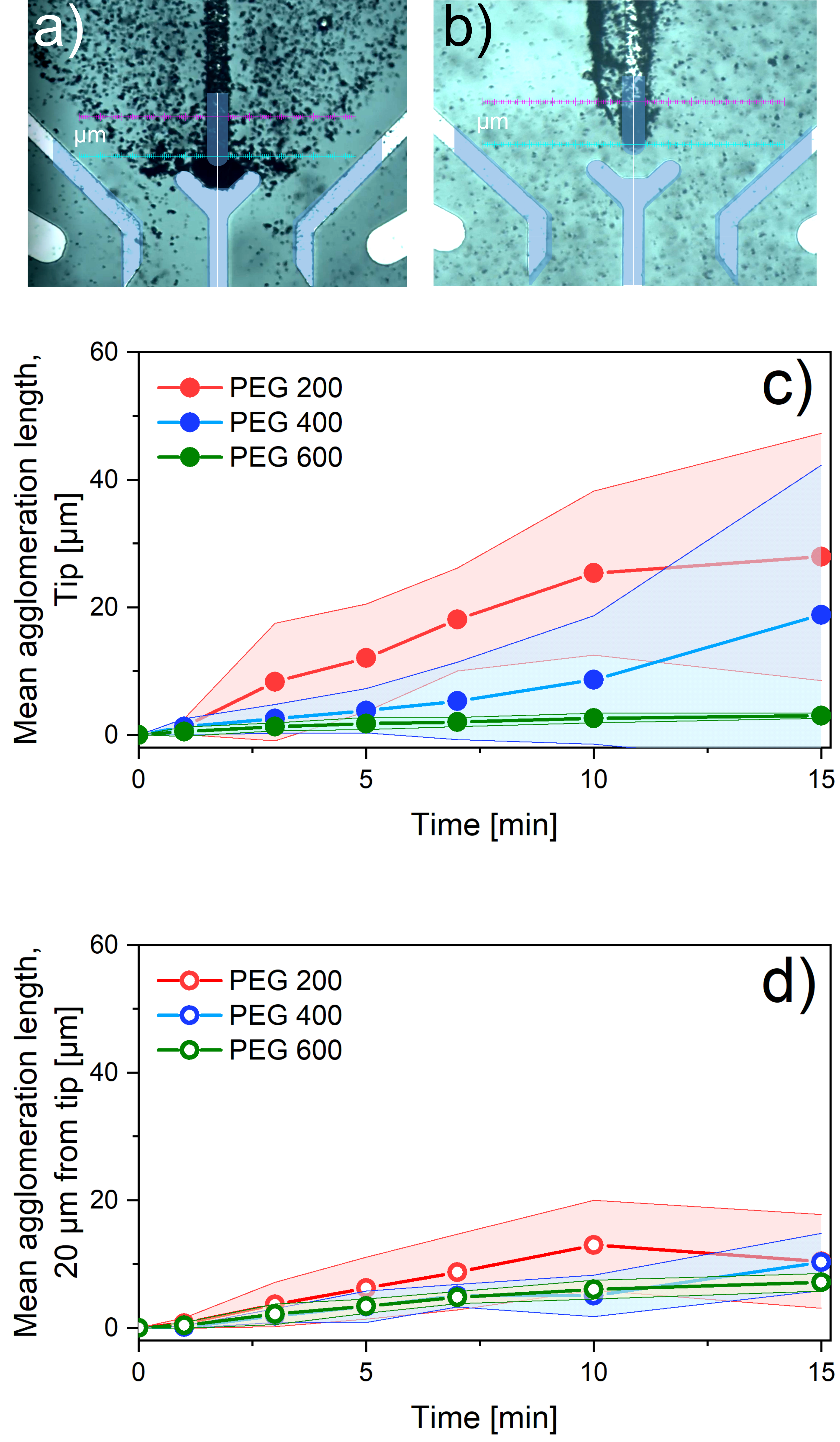


**Figure SI 7** The kinetics of the agglomeration zone growth. **a, b** The screenshots of the optical microscopy videos for Ag/PEG200 and Ag/PEG600 obtained at the 15th minute of applied constant bias overlapped with the semi-transparent mask to indicate the determination of mean agglomeration length. The time-evolution of the mean agglomeration length was measured **c** near the tip of the electrode (blue bar) and **d** 20 µm from the tip (magenta bar). V_in_ = +4 V was applied.

CFD simulations of NP movement in each PEG (**Video SI 2**) confirm that Ag NPs move fastest in PEG200 and slowest in PEG600, reflecting the differences in conductive bridge growth rates. This observation correlates perfectly with the results of the electrophoretic mobility of Ag NPs determined via the dynamic light scattering. It was found that this parameter decreases along the PEG series (PEG200, PEG400, PEG600) (refer to **Table 1**). These observations confirm that the host liquid’s viscosity significantly influences the kinetics of conductive bridge formation. Therefore, by selecting a PEG with appropriate viscosity, one can control the bridge formation process in resistive switching applications.

**Video SI 2** CFD simulations of Ag NPs movement in a constant electric field (V_in_ = +4 V) in PEG200, PEG400 and PEG600. The video is sped up by ×10. The NPs were assumed to be negatively charged. The charge was evaluated from the ξ-potential value (and NP’s surface charge, respectively) to be ~ 25 electrons per NP.

**SI 6. The role of the initialization step and its impact on resistive switching in subsequent I-V measurements**

Prior to the *I-V* hysteresis measurements, the nanofluid is initialized by applying a constant positive bias to the biased electrode (cf. *I–t* measurements, **Figure 1**). Consequently, due to the negative zeta potential of the Ag NPs and electrophoretic rearrangement, NPs and NP agglomerates are migrating towards and attaching to the positively biased electrode, resulting in the formation of an anisotropy in the distribution of Ag NPs in the interelectrode gap. In other words, the conductive bridge starts to grow from the positively biased electrode, and a large reservoir of Ag NPs and NP agglomerates is created around the biased electrode. During the I-V hysteresis measurements, at a negative polarity at the biased electrode, NPs are repelled from the large reservoir at the biased electrode, being pushed into the inter-electrode gap and contributing to the formation of a conductive bridge (SET processes). In contrast, at positive polarity at the biased electrode, NPs are attracted by the biased electrodes, depleting the inter-electrode gap and contributing to the dissolution of the conductive bridge (RESET processes).

Interestingly, the initialization of conductive bridges in nanofluids shows certain similarities to electroforming in filamentary memristive switching, which is commonly reported, e.g., for electrochemical metallization memories based on reconfigurable metal filament formation and dissolution in solid-state devices ^[2]^. In various reports of solid-state metal filament memristive devices, electroforming is required to create a proto-filament, bridging the dielectric gap between active and passive electrodes and initializing a nanoscale gap for subsequent reconfigurable metal filament formation and dissolution. While in past developments in two-terminal ECM memristive devices, the transition towards forming-free resistive switching was of particular interest, initialization in nanofluids poses an interesting direction to controllably grow long-range connections, potentially even between multiple terminals and in three dimensions. However, in future investigations, further focus has to be put on elucidating the impact of electrode geometries, initialization patterns, and nanofluid compositions on the stimulated growth of prototypical bridges.

**SI 7. The investigation of the resistive switching dynamics based on the analysis of I-V characteristics**

When the conductive bridge was pre-formed, 100 I-V characteristics were recorded for each sample, resulting in a total of 300 I-V characteristics for each nanofluidic system: Ag/PEG200, Ag/PEG400, and Ag/PEG600. **Figures SI 8** and **SI 9** present exemplary I-V cycles for Ag/PEG200 and Ag/PEG600, demonstrating all possible conductive states of the nanofluids: high resistance state (HRS), transition resistance state (TRS), and low resistance state (LRS), along with the switching events between them.

Since the current values in TRS and LRS can sometimes be of the same order of magnitude, distinguishing between these two states is challenging. Therefore, for statistical analysis, I-V sweeps were converted into R-V cycles using Ohm’s law (R = V/I). **Figure SI 10** presents representative R-V characteristics, illustrating the individual states of the system and the resistive switching events between them.

A detailed analysis of individual R-V characteristics allowed us to determine the occurrence rates of each switching event, as well as the 10th, 50th, and 90th percentiles of the switching voltage. These values are summarized in **Table SI 2**.

**Figure SI 8** The exemplary I-V characteristics showing HRS, TRS and LRS in Ag/PEG200 nanofluids

**Figure SI 9** The exemplary I-V characteristics showing HRS, TRS and LRS in Ag/PEG600 nanofluids

**Figure SI 10** The exemplary R-V characteristics demonstrating different types of switching events possible in the nanofluidic systems

**Table SI 2** Occurrence, 10^th^, 50^th^ (median) and 90^th^ percentile values of SET and RESET voltages for all types of switching events

|  | | **Ag/PEG200** | | | | **Ag/PEG400** | | | | **Ag/PEG600** | | | |
| --- | --- | --- | --- | --- | --- | --- | --- | --- | --- | --- | --- | --- | --- |
|  |  | Occurrence | Median [V] | 10^th^ percentile [V] | 90^th^ percentile [V] | Occurrence | Median [V] | 10^th^ percentile [V] | 90^th^ percentile [V] | Occurrence | Median [V] | 10^th^ percentile [V] | 90^th^ percentile [V] |
| **SET events** | HRS→LRS | 6 | -1.1 | -3.8 | 1.7 | 25 | -1.8 | -3.6 | 3.2 | 6 | -1.7 | -3.8 | 1.7 |
|  | HRS→TRS | 51 | -0.6 | -1.6 | 0.8 | 37 | -0.6 | -3.2 | 2.9 | 60 | -0.4 | -1.2 | 2.9 |
|  | TRS→LRS | 148 | -3.2 | -3.8 | -1.4 | 149 | -3.4 | -3.8 | -2.0 | 257 | -3.0 | -3.8 | -1.0 |
| **RESET events** | LRS→HRS | 8 | 1.3 | 2.5 | -3.0 | 13 | 1.9 | 3.8 | -3.6 | 12 | 1.8 | 3.6 | -3.6 |
|  | LRS→TRS | 95 | 1.3 | 1.7 | 0.8 | 158 | 1.3 | 1.9 | 0.6 | 252 | 1.7 | 3.0 | 0.8 |
|  | TRS→HRS | 38 | 2.4 | 3.8 | -3.4 | 44 | 2.3 | 3.8 | -3.4 | 55 | 2.5 | 3.2 | -3.6 |

Individual R-V characteristics were used to determine the distribution of resistive states across cycles. The procedure for automatically identifying HRS, TRS, and LRS is described here. A custom MATLAB script calculated the average resistance and its relative error over the voltage intervals +1.5 – +4 V and -1.5 – -4 V for each I-V cycle. The average resistance was computed as $\bar{R}=\frac{\sum_{i=1}^{N} R_{i}}{N}$, where R_i_ represents the resistance at each data point in the interval, N is the number of data points in the interval. These values were determined for positive and negative polarity and for increasing and decreasing voltage as follows:

*RE_pos/inc_*: 1.5 → 4.0 V

*RE_pos/dec_*: 4.0 → 1.5 V

*RE_neg/inc_*: -1.5 → -4.0 V

*RE_neg/dec_*: -4.0 → -1.5 V

For better visualization, these intervals are also shown in the representative R-V characteristic in Supporting Information (**Figure SI 11**).

Since TRS is characterized by substantial resistance fluctuations, the average resistance R̅ alone may not be sufficient to distinguish TRS from LRS. Therefore, the relative error was calculated as $RE=\frac{\sigma}{\bar{R}}*100\%$, where σ is a standard deviation, which is defined as $\sigma=\sqrt{\frac{\sum_{i=1}^{N} {(R}_{i}-\bar{R})^{2}}{N}}$. **Figure SI 12** illustrates the distributions of individual resistive states across different branches of the R-V cycles for Ag/PEG200, Ag/PEG400, and Ag/PEG600 samples.

**Figure SI 11** **a** The I-V characteristic demonstrating pinched hysteresis, characteristic of the memristive behaviour; **b** corresponding R-V characteristic with graphical representation of the branches used for the estimation of different resistive states


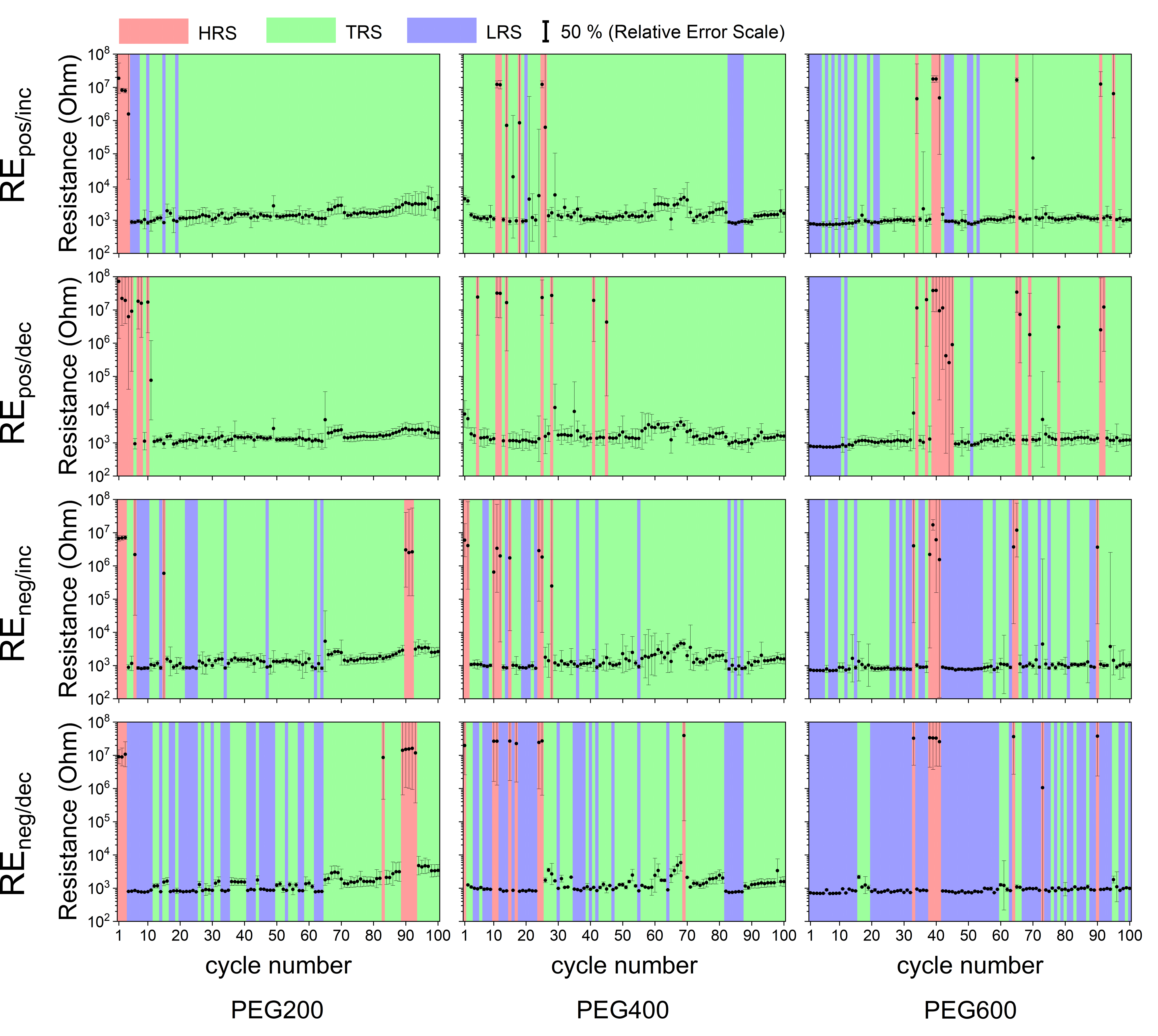


**Figure SI 12** The distribution of individual resistive states on different branches of I-V characteristics during another set of 300 cycles. The red color corresponds to HRS, green to TRS (including both types) and blue to LRS. This distribution confirms the trend described in the main article.

To accurately explain the physical origin of TRS2, the R-V characteristics were fitted while considering all possible conductivity mechanisms. Based on the literature, four mechanisms were evaluated: the hopping conduction model, Poole-Frenkel hopping, thermally assisted tunneling, and field-induced Fowler-Nordheim tunneling. The I-V equations from ref. ^[3]^ were adapted to fit the R-V characteristics. The results, illustrated in **Figure SI 13**, indicate that the field-induced Fowler-Nordheim tunneling mechanism provides the best agreement with the experimental data, making it the most suitable mechanism for describing the observed conductivity.


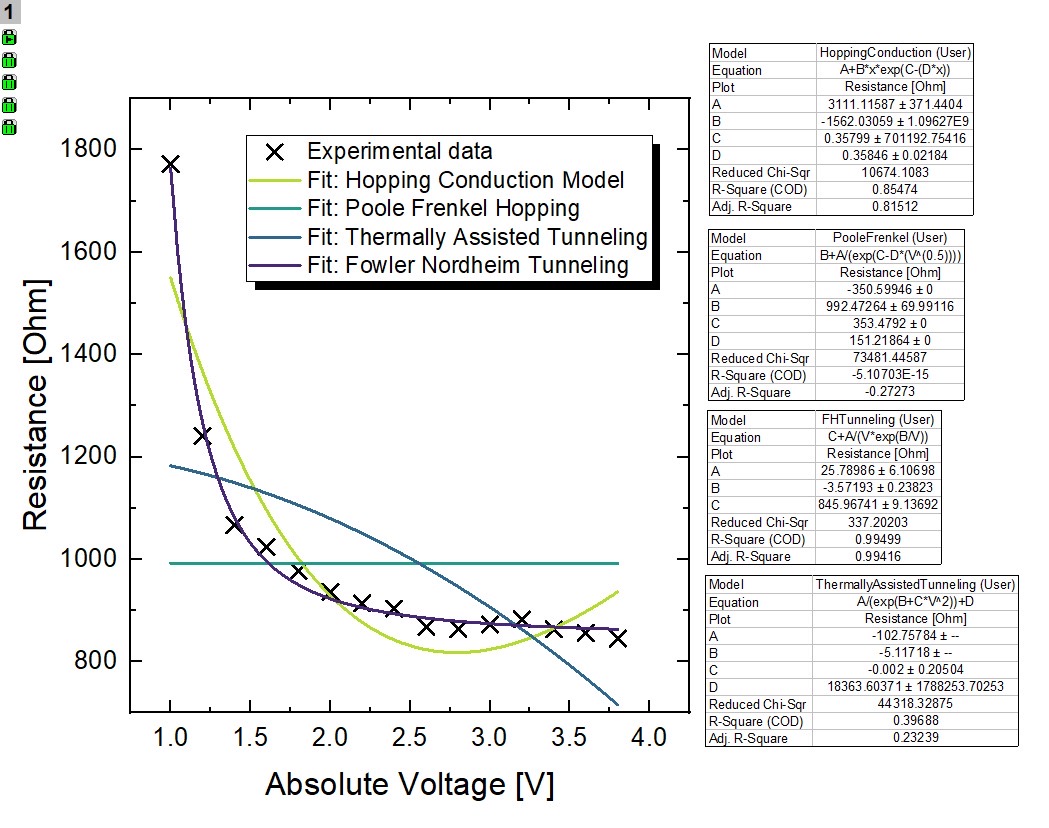


**Figure SI 13**. The fitting procedure of R-V data corresponding to TRS2 applying functions for different charge transfer mechanisms: hopping conduction model, Poole-Frenkel Hopping, thermally assisted tunneling and field-induced Fowler-Nordheim tunneling.

To obtain the cumulative occurrence plot presented in Figure 5a in the main article, the observed resistance levels from the four subsections of each of the 200 I-V hysteresis cycles for each nanofluid were sorted in ascending order. Cumulative occurrence represents the probability of observing a resistance level equal to or smaller than the respective value. The following protocol was developed to provide a more detailed analysis of individual resistive states in Ag/PEG nanofluids. All states were identified in all cycles, and afterwards, according to the number of identified states in each cycle N_cycle_∈ {1,2,3,4}, we assigned a weight corresponding to 1/N_cycle_ to the states. Subsequently, we calculate the so-called weighted fraction of individual states i, i.e., the resulting fraction is

$f_{i}=\frac{total number of states i multiplied by their weight}{total number of states}$ (3).

**SI 8. Determination of the adsorbed polymer layer characteristics**

The parameters of the macromolecular chains in the adsorbed layer on the surface of Ag NPs were evaluated based on the De Gennes formalism that relates the thickness of the adsorbed layer *L* to the grafting density *σ* (the ratio of the attached chains to the mobile chains that do not adhere to the surface) ^[4]^. The thickness is given by the relationship *L = naσ^1/2^*, where *n* is the number of monomers per polymer chain and *a* is the length of one monomer. Our previous investigation found that PEG segments were adsorbed onto NPs in a low grafting density regime, with the thickness of the adsorbed layer being very close to the characteristic size of an ideal chain *R_0_* ^[5]^. Considering that in polymer melts, *R_0_ = an^1/2^*, where *a* = 0.35 nm for PEG ^[6]^, and assuming that *L* is approximately equal to *R_0_*, we can conclude that *σ* decreases with increasing molecular mass of PEG. The results of the evaluation are represented in **Table SI 3**.

**Table SI 3** The parameters of the adsorbed polymer layer determined through the De Gennes formalism

| PEG type | *n* | *R_0_*, nm | *σ* |
| --- | --- | --- | --- |
| PEG200 | 5 | 0.8 | 0.21 |
| PEG400 | 9 | 1.1 | 0.11 |
| PEG600 | 14 | 1.3 | 0.07 |

.

**References**

[1] R. J. Sengwa, S. Choudhary, P. Dhatarwal, *J Mol Liq* **2016**, *220*, 1042, DOI 10.1016/j.molliq.2016.05.014.

[2] I. Valov, R. Waser, J. R. Jameson, M. N. Kozicki, *Nanotechnology* **2011**, *22*, 254003, DOI 10.1088/0957-4484/22/25/254003.

[3] D. Conklin, S. Nanayakkara, T.-H. Park, M. F. Lagadec, J. T. Stecher, M. J. Therien, D. A. Bonnell, *Nano Lett* **2012**, *12*, 2414, DOI 10.1021/nl300400a.

[4] P. G. de Gennes, *Macromolecules* **1980**, *13*, 1069, DOI 10.1021/ma60077a009.

[5] D. I. Nikitin, S. A. Madkour, P. L. Pleskunov, R. Tafiichuk, A. Shelemin, J. Hanuš, I. Gordeev, E. V. Sysolyatina, A. Y. Lavrikova, S. A. Ermolaeva, V. A. Titov, A. Schönhals, A. Choukourov, *Soft Matter* **2019**, *15*, 2884, DOI 10.1039/c8sm02413h.

[6] J. L. Perry, K. G. Reuter, M. P. Kai, K. P. Herlihy, S. W. Jones, J. C. Luft, M. Napier, J. E. Bear, J. M. DeSimone, *Nano Lett* **2012**, *12*, 5304, DOI 10.1021/nl302638g.
